# Contrasting packing modes for tubular assemblies in chlorosomes

**DOI:** 10.1007/s11120-024-01089-3

**Published:** 2024-03-27

**Authors:** Yuliya A. Miloslavina, Brijith Thomas, Michael Reus, Karthick Babu Sai Sankar Gupta, Gert T. Oostergetel, Loren B. Andreas, Alfred R. Holzwarth, Huub J. M. de Groot

**Affiliations:** 1 Institute of Chemistry, Leiden University, Box 9502, 2300 RA Leiden, The Netherlands; 2 Max-Planck-Institut für Multidisziplinäre Naturwissenschaften, Am Faßberg 11, 37077 Göttingen, Germany; 3 Present Address: Eduard-Zintl-Institut für Anorganische and Physikalische Chemie, Technische Universität Darmstadt, Alarich-Weiss-Str, 64287 Darmstadt, Germany; 4 Present Address: Science Division, New York University Abu Dhabi, P.O. Box 129188, Abu Dhabi, United Arab Emirates; 5 Max-Planck-Institut für Chemische Energiekonversion, Stiftstr. 34-36, Mülheim a. d. Ruhr, Germany; 6 Groningen Biomolecular Sciences and Biotechnology Institute, University of Groningen, 9747 AG Groningen, The Netherlands; 7 Present Address: Department of Physics and Astronomy, Faculty of Sciences, Vrije Universiteit Amsterdam, Amsterdam, The Netherlands

**Keywords:** Chlorosomes, Solid-state NMR, Structure, Light harvesting, Photosynthesis, *Chlorobaculum tepidum*

## Abstract

The largest light-harvesting antenna in nature, the chlorosome, is a heterogeneous helical BChl self-assembly that has evolved in green bacteria to harvest light for performing photosynthesis in low-light environments. Guided by NMR chemical shifts and distance constraints for *Chlorobaculum tepidum* wild-type chlorosomes, the two contrasting packing modes for *syn-anti* parallel stacks of BChl *c* to form polar 2D arrays, with dipole moments adding up, are explored. Layered assemblies were optimized using local orbital density functional and plane wave pseudopotential methods. The packing mode with the lowest energy contains *syn-anti* and *anti-syn* H-bonding between stacks. It can accommodate *R* and *S* epimers, and side chain variability. For this packing, a match with the available EM data on the subunit axial repeat and optical data is obtained with multiple concentric cylinders for a rolling vector with the stacks running at an angle of 21° to the cylinder axis and with the BChl dipole moments running at an angle *ß* ∼ 55° to the tube axis, in accordance with optical data. A packing mode involving alternating *syn* and *anti* parallel stacks that is at variance with EM appears higher in energy. A weak cross-peak at -6 ppm in the MAS NMR with 50 kHz spinning, assigned to C-18^1^, matches the shift of antiparallel dimers, which possibly reflects a minor impurity-type fraction in the self-assembled BChl *c*.

**Supplementary Information:**

The online version contains supplementary material available at 10.1007/s11120-024-01089-3.

## Introduction

Chlorosomes perform rapid and efficient excitation energy transfer (EET) across distances of 100 nm (Dostal et al. 2012; Prokhorenko et al. 2000). They contain up to several hundred thousand bacteriochlorophylls (BChls) that form their responsive matrix for light harvesting by modulated self-assembly (Holzwarth et al. 1990). In contrast to other photosynthetic antennae, for which a protein environment interacts with the cofactor molecules to reinforce the biological function, every chlorosome is different in size and overall shape. They present a unique opportunity to determine how a chiral responsive matrix of biological origin with very limited complexity can establish a low loss long-range energy transfer mechanism by rapid quasi-coherent interconversion of exciton states induced by thermal motion (Purchase and de Groot 2015; Sawaya et al. 2015; Li et al. 2020) and can serve as a blueprint for the bottom-up chemical design of artificial light-harvesting systems (Katterle et al. 2007).

The BChls in chlorosomes are thought to form *syn-anti* parallel stacks that self-assemble in polar chiral sheets, and concentric tubes with varying curvature where the dipole moments are partially averaged over the circumference of the cylinders and a net dipole moment is established for the light harvesting function (van Rossum et al. 2001; Ganapathy et al. 2009; Günther et al. 2016; Li et al. 2018; Eric et al. 2023a). Both ^13^C Magic Angle Spinning (MAS) NMR and optical spectroscopy have revealed two distinct spectral fractions with remarkably small inhomogeneous linewidths approaching ∼ 1 ppm in the ^13^C NMR and ∼ 100 cm^−1^ in optical data (Balaban et al. 1995; Psencik et al. 1994; Günther et al. 2016). The ratios of the two components in MAS NMR data collected from WT, *bchQRU*, and *bchQR* were 7:3, 9:1, and 1:1, respectively (Ganapathy et al. 2009; Ganapathy et al. 2012; Balaban et al. 1995). The major homolog from the WT (wild type), the 17^2^-farnesyl-(*R*)-[8-ethyl,12-ethyl]BChl *c*, yields a ^13^C MAS NMR response after self-aggregation in hexane that is very similar to the mixture for the WT, including the spectral doubling (Balaban et al. 1995).

Recent atomistic simulations of the chlorosome self-assembly have associated the two structural fractions with a bimodal distribution in a tubular plastic crystal phase (Li et al. 2022). The packing allows for restrained molecular rotational dynamics in the BChl planes on the ps timescale involving switching between 0 and 1 or 1 and 2 interstack hydrogen bonds (Li et al. 2020, 2018; Li et al. 2022). For the first configuration, the dynamic equilibrium is shifted towards one H-bond, while for the second part, it is biased towards two interstack H-bonds. Due to motional averaging narrow MAS NMR signals are observed and the two spectral fractions are thought to correspond with H-bond donors and non-donors (Eric et al. 2023b). In order to form the interstack H-bonds the BChl have to undergo a staggered in plane rotation by ca +/− 20 degrees. This leads to a frustrated system where the H-bonding pattern is essentially randomized over the self-assembly.

In parallel, for various chlorosome species a narrow inhomogeneous linewidth profile of ∼ 100 cm^−1^ was determined with hole burning from the zero-phonon hole action spectrum at the low energy side of the optical absorption, collected at low temperature to quench energetically uphill transfer into the exciton manifold of states and narrow the absorption profile (Fetisova and Mauring 1993; Psencik et al. 1994). Optical linewidths down to 100 cm^−1^, have been corroborated by analyses of single chlorosome fluorescence excitation spectra (Günther et al. 2016). The manifold of higher exciton states is dispersed by the bending of BChl layers (Tian et al. 2011; Günther et al. 2016). The distinction between homogeneous and inhomogeneous broadening vanishes, as ultrafast delocalization affects the entire absorption profile (Fetisova and Mauring 1993, 1992; Fetisova et al. 1996, 1994). Quantum dynamics simulations provide insight into how ultrafast EET delocalization can be induced by the libration dynamics. The rotational sampling of H-bonding configurations of the BChls modulates the π−π overlap between the BChl macrocycles, which gives rise to frequent and abundant sweeps of levels over avoided crossings across the manifold of exciton states for rapid redistribution of exciton density over the entire chlorosome (Li et al. 2020). The balance between inhomogeneous dispersion of exciton states and homogeneous thermally induced delocalization, due to rotational dynamics of the BChl, is an example of how life generates and exploits apparent paradoxes for biological function. It allows for overcoming the localization of excitons in the heterogeneous chlorosome environment by a crossover to delocalization of optically active states and superradiance from the distribution of the excitation over the tubular geometry (Molina et al. 2016; Li et al. 2020). While static heterogeneity suppresses exciton delocalization, thermally activated dynamic disorder generates transient off-diagonal terms for rapid non-adiabatic delocalization of excitons. (Haken and Reineker 1972; Huelga and Plenio 2013; Sawaya et al. 2015; Li et al. 2020). Stochastic fluctuations from random thermal motion in the structure lead to coupled coherent-incoherent mixing of exciton states for the distribution of exciton density over the concentric tube self-assemblies (Li et al. 2020).

**Fig. 1:**
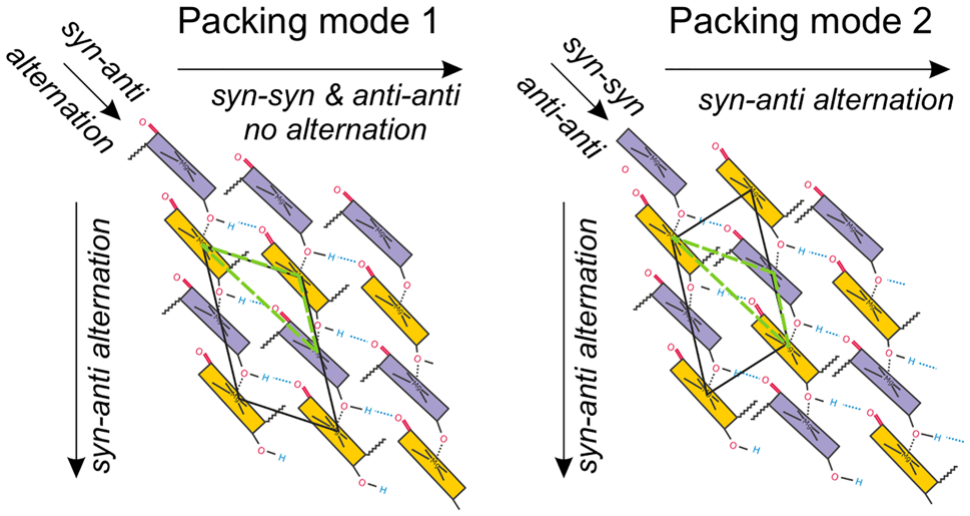
Possible interstack arrangements of *syn-anti* parallel stacks to form a 2D sheet, as in the chlorosome structure. Schematic patterns of *syn* (purple) and *anti* (yellow) BChls. When alternating *syn-* and *anti*-conformers are packed into a 2D array, this leads to a triangular lattice topology where in two directions there is alternation, while in the third direction, the sequence is all-*syn* or all-*anti* (green triangles)

Determining the packing of BChls in the intrinsically heterogeneous chlorosomes, underlying their unique harvesting properties, requires a dedicated approach. For a *Chlorobaculum (Cba.) tepidum bchQRU* mutant with reduced heterogeneity, ∼ 90% of the BChl is of the H-bond donor type. By projection of the heterogeneous structure on an idealized, homogeneous concentric tube model with helical symmetry, *syn-anti* stacking of the BChl could be resolved by MAS NMR ^13^C–^13^C homonuclear and ^1^H–^13^C heteronuclear dipolar correlation spectroscopy, in combination with Fourier transform (FT) cryo-EM. Within a hybrid structure determination approach, a 2D cryo-EM periodogram, generated from high-resolution cryo-EM images by Fourier analysis, was used to resolve the spacing between cylinders and to determine an 8.3 Å subunit axial translation, pointing to *syn-anti* parallel stacks forming rings that self-assemble in concentric tubes with a multi-start helical BChl supramolecular arrangement (Ganapathy et al. 2009). Here *syn* means that the out-of-plane doming of the Mg^2+^ ion and the C-17^1^ atom from the tail are both on the same side of the porphyrin ring (Fig. 1, S5, S6), while *anti* means that they are on opposite sides (van Rossum et al. 2001). The BChls are connected by a Mg^2+^⋅⋅⋅O–H⋅⋅⋅O=C recognition motif with strong π−π overlap along the *syn-anti* parallel stacks for optical excitation.

Wild type chlorosomes are more heterogeneous than for the *bchQRU* mutant, due to variations in the 8 and 12 side chains and a larger fraction, ∼ 30%, of non-donor BChl induced by the packing. MAS NMR ^1^H and ^13^C ring-current shifts, together with internuclear ^1^H–^13^C and ^13^C–^13^C correlations have also revealed *syn-anti* parallel stacks of BChls (Ganapathy et al. 2009). According to cryo-EM, they self-assemble into multi-start helices with a 12.5 Å subunit axial translation and further into concentric tubes with a 20–21 Å radial repeat (Ganapathy et al. 2009). In contrast with the *bchQRU* mutant, where the stacks are running roughly perpendicular to the tube axis and form rings or helices with a gentle slope, the stacks in the WT chlorosomes are running roughly in the direction of the cylinder axis forming much steeper helices (Ganapathy et al. 2009). According to single-molecule optical spectroscopy, the angle between the stacks and the cylinder axis is ∼ 20° (Gunther et al. 2018).

Despite the obvious differences between the various chlorosomes at the supramolecular level, the packing into sheets appears robust, and insensitive to molecular details and heterogeneity (Li et al. 2022). In particular, the quasi-2D interaction network is only mildly deformed by variation in H-bonded and non-H-bonded fractions, and curvature of the sheets. It can accommodate varying ratios of *R* and *S* diastereoisomers and different homologs in the same robust framework with alternating coaxial regions of tails and overlapping rings. The specific aim of this study is now to use the previously established, idealized planar 2D sheet model for the major, H-bonded BChl component to analyze the interstack arrangement for achieving an optimal packing with *syn-anti* pseudosymmetry at the molecular level. For this purpose, MAS NMR structural data are collected from WT *Cba. tepidum* chlorosomes with reduced compositional heterogeneity, which is the result of growing the organism in high light. This leads to a more homogeneous structure with less variation in the side chains than for previous preparations (Tian et al. 2011). We recorded HETCOR, RFDR, and hChhCh data from ^13^C-labeled samples with 30 kHz MAS frequency, and obtained a much better spectral resolution and more intermolecular correlations, compared to our earlier studies. A very similar ratio of 7:3 between H-bond donor and non-donor BChl fractions is observed from the doubling of the C-5 and C-7^1^ cross-peaks as for the earlier preparations. This corroborates the earlier findings that the doubling of signals is an intrinsic property. With pseudosymmetric *syn-anti* pairs forming parallel stacks with staggered optical transition dipoles, there are two different ways to form sheets, either by forming *syn-anti* and *anti-syn* interstack H-bonds or by forming *syn-syn* and *anti-anti* interstack H-bonds (Fig. 1). The pseudo symmetric building block is an example of an induced misfit in the form of the *syn-anti* arrangement at the basis, which propagates into higher levels of the chlorosome structural hierarchy. From a biological perspective, it provides diversification by molecular control at the level of the BChls for evolutionary selection between distinct possible arrangements. Guided by NMR data, we identify which of those two interstack arrangements appears most appropriate to describe the BChl self-assembly mode for WT.

## Materials and methods

### Chlorosome preparation

Green sulphur bacteria WT *Cba. tepidum* was grown anaerobically in the high-light condition to reduce heterogeneity (Fig. S1). Fluorescence tubes (Osram, a mixture of 18W/25 universal-white and 18W/77 Fluora) were used with an illumination intensity of 125 µE/(s⋅m^2^) at the surface of 1.5 L continuously stirred reactor bottles. Growth of the bacteria was over several generations on ^13^C-labeled bicarbonate. The growth was stopped at relatively low cell density to minimize self-shading effects. Isolation was as in Reference (Tian et al. 2011), except for the last step. Before RFDR experiments the chlorosomes were washed with D_2_O containing buffer to suppress possible long-range transfer from residual solvent. For the HCH and hChhCH experiments, the sample was not washed with D_2_O. After the concentration of chlorosomes at ∼ 200000 × *g* for one hour in an ultracentrifuge, the rotor was filled as a chlorosomes paste. The purification method was generally much milder than before (Ganapathy et al. 2009), using NaSCN instead of LDAO.

### Solid-state NMR measurements

MAS NMR experiments were performed as described in Fig. S2 on freshly prepared homogeneously ^13^C-labeled *Cba. tepidum* WT chlorosome preparations. For all measurements the sample was at 277–278 K in the state of a paste. For the data collected at 750 MHz we used 30 kHz spinning. The temperature effects were moderate, and the temperatures are accurate to ∼ 1 degree. For the data collected at 800 MHz we used faster spinning. At 50 kHz the temperature was calibrated using external KBr measured at the same conditions as chlorosomes.

**Fig. 2:**
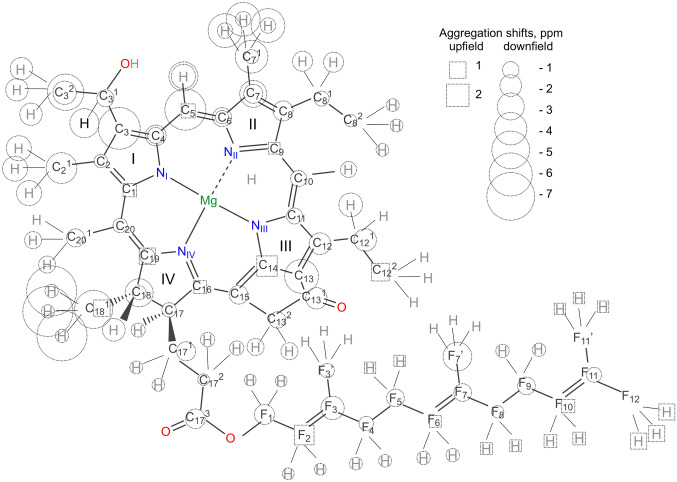
The most abundant homolog of *Chlorobaculum* (*Cba.) tepidum* [8-Et, 12-Et]-BChl *c*. Other homologs have different methylated substituents at carbons 8, 12 and varying *R*/*S* epimer ratio at 3^1^. Up-field aggregation shifts for chlorosomes are marked as circles, downfield as squares. The sizes of circles and squares are proportional to the magnitude of the aggregation shifts in Tables S1, S2

Before setting up the experiments on chlorosomes, chemical shifts were calibrated by known chemical shifts of solid tyrosine in Leiden or adamantane in Göttingen, on which also pulse sequences were tested. The carbon shifts were externally referenced to TMS and we used the frequency relation between protons and carbons to arrive at the proton shifts.

**Fig. 3:**
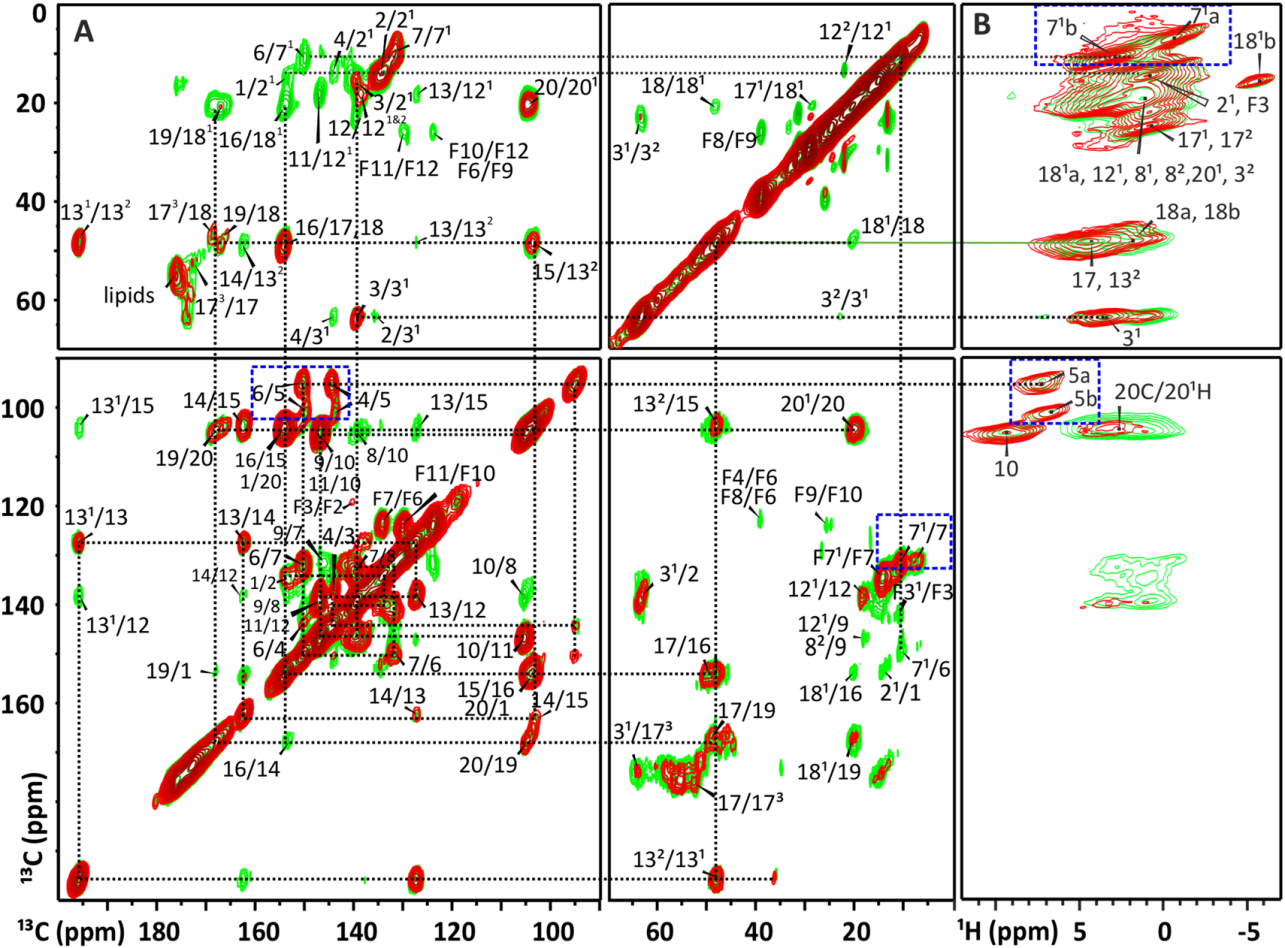
^13^C–^13^C RFDR (**A**) and cross-polarization-based ^1^H–^13^C (**B**) spectra of WT *Cba*. *tepidum* with assignments. The lines indicate the nearest neighbor correlations. The RFDR data were recorded with 1.0 (red) and 2.9 (green) ms mixing times at the 750 MHz spectrometer at 30 kHz MAS. The ^1^H-^13^C was recorded with proton detection with 0.5 (red) and 3 (green) ms of carbon to proton cross polarization at the 800 MHz magnet with 50 kHz MAS (also see Fig. S7 for the HCH spectra with less contours). The region from 70 to 90 ppm does not contribute to the BChl NMR response and was left out. The most pronounced doublings are marked by blue rectangles, a and b mark the major and minor doubling component that can be attributed to H-bonded and non-H-bonded BChls, respectively

The BChl *c* molecule contains 50 non-equivalent carbon atoms (Fig. 2). Among them are 16 quaternary carbons on the ring, and 3 in the tail, also 7 tertiary, 10 secondary, and 10 primary carbons, one alcohol-, one keto- and one carboxylic group. The more electronegative the environment around a ^13^C is, the more its NMR response is shifted downfield, leaving C-13^1^ and C-17^3^ groups as the most downfield in the spectra (Figs. 2, 3, Tables S1-S2). Carbons and protons that are positioned above the porphyrin ring of a neighboring BChl experience significant ring-current shifts (Abraham and Smith 1983; Abraham et al. 1982; Ema et al. 2005).

2D ^13^C−^13^C RFDR (Fig. S2A) was performed in a 2.5 mm rotor spinning at 30 kHz at the magic angle in a Bruker AV-750 spectrometer (Karlsruhe, Germany) equipped with a double resonance probe. For each of the 1536 steps in the indirect ^1^H dimension, 128 ^13^C scans were collected. To probe the ^13^C network, RFDR data (Bennett et al. 1995) were collected with mixing times of 0.5, 1, 1.4, 2.9, and 4.3 ms (first and last are shown in Fig. 3A). The assignments of the carbon chemical shifts were validated first by the RFDR experiment with a short mixing time of 1 ms (Fig. 3A, red color). The correlation signals are predominantly associated with the transfer of polarization between neighboring carbons, and the polarization transfer from one carbon to the other in the ^13^C network of the BChl *c* is marked by the dotted lines. Table S1 comprises the solid-state chemical shifts ${\sigma }_{\text{s}}^{\text{C}}$, the shifts for the monomeric BChl *c* in solution ${\sigma }_{\text{l}\text{i}\text{q}}^{\text{C}}$, and the aggregation shifts${\varDelta \sigma }_{\text{s}}^{\text{C}}$, which mark the differences between the solid state and solution. At a longer mixing time of 3 ms (Fig. 3A, green), the extended range of polarization transfer will allow for the detection of longer-range correlations between distant ^13^C–^13^C over 2 or 3 bonds from the relayed transfer.

^1^H–^13^C HCH (Fig. S2B) experiments were performed in a 1.3 mm rotor spinning at 50 kHz MAS in a Bruker AVIII-800 spectrometer (Karlsruhe, Germany) in Göttingen with short 0.5 and long 3 ms mixing times to obtain assignments of protons ${\sigma }_{\text{s}}^{\text{H}}$ (Table S2). To assign the proton response in the HCH data (Fig. 3B), the RFDR data for ^13^C nuclei were used. The ^1^H chemical shift scale was internally calibrated using the water signal and the known temperature as measured with KBr. A ^1^H π/2 pulse length of 2.5 µs was used with cross-polarization periods of 1.5 and 0.5/3 ms. For each of the 1056 steps in the indirect dimension, 4 scans were collected.

**Fig. 4:**
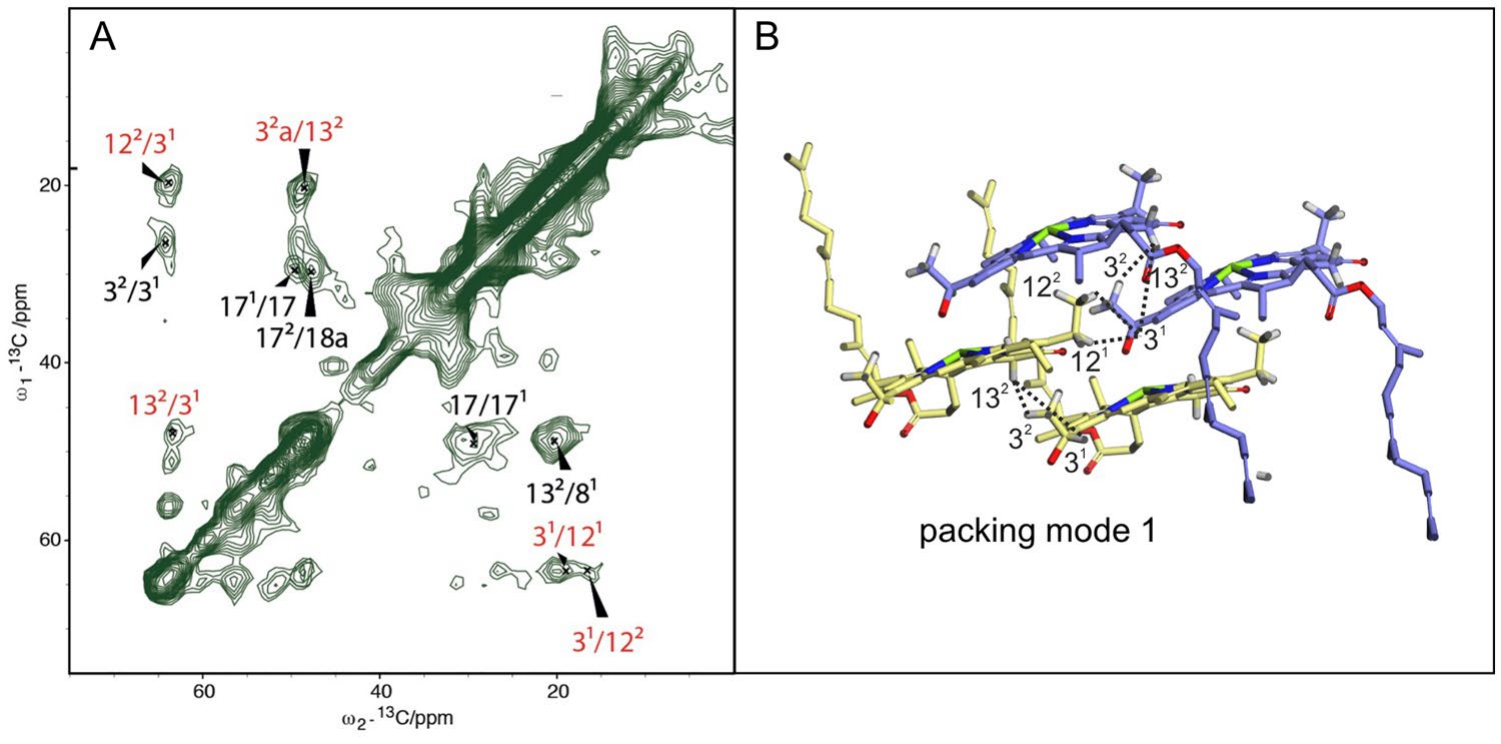
^13^C–^13^C RFDR (**A**) and cross-polarization-based ^1^H–^13^C (**B**) spectra of WT *Cba*. *tepidum* with assignments. The lines indicate the nearest neighbor correlations. The RFDR data were recorded with 1.0 (red) and 2.9 (green) ms mixing times at the 750 MHz spectrometer at 30 kHz MAS. The ^1^H-^13^C was recorded with proton detection with 0.5 (red) and 3 (green) ms of carbon to proton cross polarization at the 800 MHz magnet with 50 kHz MAS (also see Fig. S7 for the HCH spectra with less contours). The region from 70 to 90 ppm does not contribute to the BChl NMR response and was left out. The most pronounced doublings are marked by blue rectangles, a and b mark the major and minor doubling component that can be attributed to H-bonded and non-H-bonded BChls, respectively. Fig. S7 gives a detailed view at high resolution

3D ^13^C−^13^C hChhCH (Fig. S2C) experiments were performed in a 1.3 mm rotor with 50 kHz MAS in the same equipment as ^1^H–^13^C HCH. This sequence was used for the indirect detection of ^1^H−^1^H contacts with CP contact times, set to 300 µs to ensure the direct polarization transfer restricted to ^1^H–^13^C spin pairs. For each of the 188 steps in the indirect dimension, 2 scans were collected. The hChhCH was used to detect intermolecular correlations for ^13^C–^1^H transfers and to elucidate the 3D structural arrangements (Aluas et al. 2009). The distance constraints obtained from MAS NMR were compared with distances from geometry optimization of the various models (Fig. 4).

### Modeling

Geometry optimization DMol^3^ allows the simulation of the electronic structure, charges, and energetics of molecules using DFT (Delley 1996). This method produces accurate results while keeping the computational cost reasonable for an *ab initio* method. CASTEP employs the DFT plane-wave pseudopotential method for the calculation of NMR chemical shifts (Clark et al. 2005; Bonhomme et al. 2012). Before quantum mechanical calculations, starting structures were prepared with the Forcite collection of molecular mechanics tools (Mayo et al. 1990). The Dreiding force field within Forcite based on hybridization rules was modified to account for the Mg^2+^ ion. It was added manually in a way similar to Zn^2+^. The distances and angles were taken from a crystal structure of ethyl chlorophyllide *a* (Chow et al. 1975). The Particle-Particle Particle-Mesh method (PPPM) of summation was used for the electrostatic interactions, the Ewald technique for the van der Waals terms (Ewald 1921), and atom summation for hydrogen bonding terms. The PPPM uses a Discrete FT to evaluate the reciprocal space part of the Ewald sum, which for large systems can be significantly faster than the ordinary Ewald sum (Hockney and Eastwood, 1988).

For DMol^3^ geometry optimization, the exchange-correlation energy Perdew-Burke-Ernzerhof (PBE) functional of the generalized gradient approximation (GGA) was used (Perdew et al. 1996) with a self-consistent field tolerance of 1.0^−5^ eV per atom.

For both geometry optimization and chemical shift calculations in CASTEP, the Generalized Gradient Approximation (GGA) was used with the exchange-correlation functional of PBESOL using a plane-wave energy cutoff of 10.0 eV. Calculated chemical shielding *δ*_S_ was converted into chemical shifts *σ*_S_^C^ using the relation *σ*_S_^C^ *= σ*_ref_ −* δ*_S_. Here the *σ*_ref_ are determined by a linear regression between calculated and experimental shifts for the proposed packing. The electronic minimization method used for the self-consistent field calculation was density mixing with a self-consistent field tolerance of 1.0^−5^ eV per atom (Clark et al. 2005; Baias et al. 2013). Simulations were run under atmospheric pressure at room temperature with Grimme DFT-D correction.

The CrystalMaker software package was used to bend BChl surfaces based on different packing modes into tubes. Tubes were also gathered together within this software.

Software EMAN2 was used to simulate the Electron Microscopy (EM) projection of the tubes (Fig. S4).

## Results

### Radiofrequency-driven dipolar recoupling (RFDR) and heteronuclear decoupling

Combined homonuclear ^13^C–-^13^C and heteronuclear ^1^H–-^13^C data were collected at a ^13^C frequency of 180 MHz with 30 kHz MAS from the optically homogeneous sample grown in high light (Fig. 3). The resolution is significantly improved compared to our earlier WT studies that were performed with 11 kHz MAS on chlorosomes grown in low light intensity (Balaban et al. 1995; Ganapathy et al. 2012). The degree of alkylation of BChl side chain substituents is inversely proportional to the light intensity during growth (Huster and Smith 1990; Borrego et al. 1997). While compositional heterogeneity broadens the absorbance of chlorosomes for better growth when light is limiting, it introduces static inhomogeneity on the molecular level that will be visible in the NMR as multiple signals. In the highly resolved RFDR data for the present preparation with reduced compositional heterogeneity from cells grown in high light, distinct C-9, C-16, and C-17 responses are detected (Gomez Maqueo Chew et al. 2007; van Rossum et al. 2001; Ganapathy et al. 2012). In addition, the carbons at positions 3, 3^1^, 10, 11, 12, 13^1^, 13^2^, 14, and 15 have well-resolved cross-peaks that appear as unique signals. In contrast, the doubling attributed to the presence of H-bonded and non-H-bonded BChl appears independent of the compositional heterogeneity. The data in Fig. 3 confirm that the ratio of principal doubling of ∼ 7:3 in major and minor components for the C-5 and C-7^1^ signals is an intrinsic property of the WT chlorosome structure (Ganapathy et al. 2012; Luo et al. 2014). The minor doublings for C-4, C-8, and C-18 are also better resolved in comparison to previous measurements (van Rossum et al. 2001; Ganapathy et al. 2012). At 50 kHz spinning frequency, additional doublings were observed for the C-2^1^, 3^1^, 3^2^, 17^3^, and for the H-18 proton signals that were not resolved earlier. Correlated inhomogeneity is observed in the upfield proton cross-peak at around − 6 ppm (Table S2 and Fig. S7) corresponding to C-18 resonances at 45 and 48 ppm carbon. An additional correlation at − 6 ppm on proton with a 15–16 ppm resonance for carbon, was assigned to C-18^1^ of the non-H-bonded BChl, representing a minor fraction of a doubled response (Li et al. 2018, 2020; Eric et al. 2023b).

The ^13^C in the tails show stronger cross-peaks than for previously measured RFDR spectra. In particular, the intensities of the F6/F7 and F10/F11 correlations between carbons in the tail are well visible. Although carotenoid ^13^C also resonates around 120 ppm, strong correlation signals contributing to the intensity are unlikely to originate from carotenoids, which represent < 10% of the molecular constituents in chlorosomes (Adams et al. 2013). F2 has a resolved cross-peak that disappears at longer, 3 ms, mixing time. The F5/F9 and F4/F8 correlation signals overlap. A strong F7/F7’ response is likely combined with the C-2/C-2^1^ correlation signal.

### Construction of a packing mode 1 from the repeat unit 1

To model the packing, periodic systems were constructed from a repeat unit containing a pair of pseudosymmetric BChls in the *syn-* and *anti-* configuration (See Supplementary materials for details on the construction of a repeat unit). This led to extended sheets of interacting stacks in 3D packings that were constructed starting from the average distance of 21 Å between tubes measured by EM for WT chlorosomes (Ganapathy et al. 2009). After *ab initio* geometry optimization with a plane wave basis set a refined repeat unit 1 was obtained (Table S4). The *a*-axis was elongated relative to the initial packing model for the WT derived from the *bchQRU* system (Ganapathy et al. 2009). The distance between monomers along the stack varied the most, while the *b*-axis varied much less when the repeat unit was optimized (Table S4). The dissection of the total energy into its contributions showed that BChls were stabilized mainly by electrostatic interactions, from Mg^2+^ coordination and from π-π interactions between the macrocycles, while hydrogen bonds between the stacks had a minor contribution (Table S9).

### Stereoisomers and homologs

Chlorosomes contain variable mixtures of *R-* and *S-* stereoisomers at physiological temperatures (Gomez Maqueo Chew and Bryant 2007). The refined repeat unit 1 was packed with diastereoisomers and ethyl- (Et) or propyl- (Pr) homologs of BChl *c* to investigate if a preference for *S-* or *R-* exists at the C-3^1^ stereo-center, and to address the effect of compositional heterogeneity in the substituents at positions C-8 and C-12. The total energies obtained with DFT local orbital calculations and with the high accuracy plane wave method for the homodimeric *SS* and *RR* and heterodimeric *RS* and *SR syn*-*anti* pairs for the [8-Et,12-Et] and [8-Pr,12-Et] (Balaban et al. 1995) were remarkably similar (Table S3).

### Packing mode 2 and its repeat unit 2

Based on the repeat unit 1 in Fig. 1B, the related parallel repeat unit 2 arrangement was constructed using regular planar arrays filled with *syn-anti* building blocks with H-bond interactions (see Fig. S6 in SI for details). For the repeat unit 2 a different pattern of *syn-(S)* and *anti*-(*R*)*-*BChls in the layers and tubes was generated while preserving the Mg-Mg distances between BChl *c* molecules.

Packing mode 2 has the same *syn-anti* types of stacks as packing mode 1, but every second stack is moved one position up. While the densities of repeat unit 1 and 2 are comparable, the two types of interstack H-bonds for packing mode 2 are different, with distances of 1.9 and 6.0 Å, in comparison to the distances in packing mode 1 that are similar, 1.7 and 2.0 Å. This might be explained by misalignment of hydrogen bonds between *syn-syn* and *anti-anti* BChls, in comparison to the hydrogen bonds in packing mode 1 between *syn-* and *anti-*BChls of adjacent stacks (Fig. 1). For BChl, the transition dipole moment runs to a good approximation along the H-bonds from the C-13^1^ keto functionality to the OH on the C-3^1^, and parallel stacking allows for extended transition dipole moments in lamellar and tubular assemblies.

### Other packing modes

Apart from the parallel packing modes 1 and 2 depicted in Fig. 1, two additional arrangements were constructed, an alternating *syn* and *anti* stacks mode with alternating parallel stacks of *syn* and parallel stacks of *anti* BChl and a possible antiparallel fraction (both are discussed in SI). The packing mode 2, alternating *syn* and *anti* stacks and the possible antiparallel dimer fraction have somewhat higher energies of 4, 1.2, and 0.3 eV, respectively, as compared to the packing mode 1 periodic lattice (E_stacking1_= − 26797.2 eV, E_stacking2_ = − 26796.0 eV, E_alternating_ = − 26793.2 eV, E_antiparallel_ = − 26796.9 eV). The energies vary only moderately, and the alternating *syn* and *anti* stacks packing mode was reported for a different mutant, *bchQR*, with half of the BChl *c* H-bonded and the other half non-H-bonded (Ganapathy et al. 2012). Since it requires alternating *syn* and *anti* stacks, this mode is at variance with NMR and EM for the WT, however. The ^13^C and ^1^H NMR chemical shifts were calculated for the optimized structures (Tables S5-8), and for the packing mode 1 the smallest root mean square deviation from the experimental data (RMSD) was found.

### Intermolecular correlations

While hChhCH intermolecular transfer events for chlorosomes are rare and difficult to detect, with few ^1^H–^1^H intermolecular distances less than 5Å between nearby substituents in the cylindrical chlorosome framework, they are significant to validate the packing of the unsaturated BChl *c* macrocycles (Ganapathy et al. 2009).

The 3D hChhCH data result in multiple intermolecular correlations for the C-3^1^ with C-12^1^, 12^2^, and 13^2^, and for the C-3^2^ with C-13^2^ (Fig. 4). These correlations validate the packing mode 1 and correspond to distance constraints between 4.2 and 4.7 Å (Fig. 4B). In contrast, for the other models these correlations would imply transfer over long distances > 5 Å, which is less likely. Some fraction of antiparallel dimers can be invoked to explain the weak − 6 ppm cross-peak assigned for the C-18^1^ methyl protons. This carbon is close to Mg^2+^ ion only in the antiparallel piggy-back packing. In parallel stacks, the C-18^1^ is on the periphery, where there is minimal ring current shift. Other doublings, in particular around 5-C, are an intrinsic property of the parallel stack model and have been recently attributed to variations in the hydrogen bonding pattern of the BChl both for WT and mutant chlorosomes (Li et al. 2018). In the Supplementary material, cif files for the optimized packing modes 1, 2 and alternating *syn* and *anti* stacks are provided.

### Construction of chlorosome tubes

**Fig. 5:**
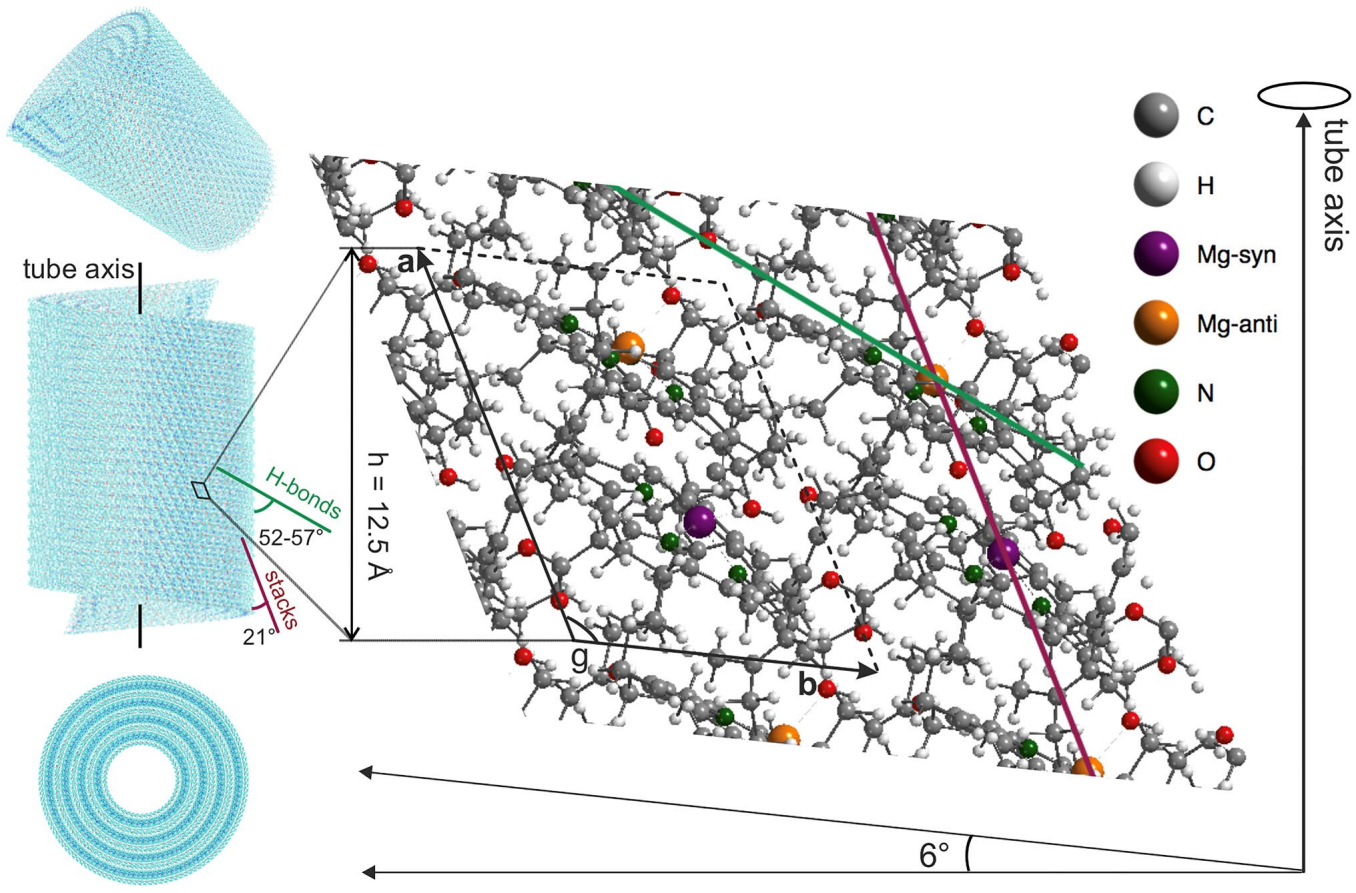
The packing mode 1 based surface with *a*, *b* axes cf. Fig 4 can be rolled into cylinders to form genuine tubular assemblies. *Syn-anti* stacks (red line) are rolled with 21° down towards the tube axis, in line with EM, NMR and optical data, and modeling. The rolling vector is defined ∼ 6° up from the *b* axis, perpendicular to the tube axis and equals to 13 primitive repeat units 1 in length. For H-bonds along *anti*-BChl, their angle with the main chlorosomal axis is 57°, and for H-bonds along *syn*-BChl the angle is 52°. The radii of the tubes are 38.6, 57.9, 77.2, and 96.5 Å

For the packing in packing mode 1, which showed the best correspondence to the NMR and modeling data, two seamless concentric tube models were constructed from four sheets bent into cylinders with radii 38.6, 57.9, 77.2, and 96.5 Å (Fig. 5) to match both the diffuse EM layer line at 1/12.5 Å^−1^ and the perpendicular equatorial reflections at 1/21 Å^−1^ (Ganapathy et al. 2009) (Fig. S4). For *a* = 13.4 Å, the experimentally observed subunit axial repeat of 12.5 Å is reproduced with a 21° tilt of the *a*-axis away from the cylinder axis. The angle of the molecular transition dipoles to the tube axis was determined as *β* = 55° from the ratio of the oscillator strengths of the exciton transitions polarized parallel and perpendicular to the cylinder axis (Günther et al. 2016). For this configuration, the best match to both the optical and EM data (Fig. S4) with concentric cylinders was obtained with a slightly modified repeat unit 1 with cell parameters *a* = 13.4 Å, b* = 9.77 Å, *c* = 22.7 Å with angles 90°, 90°, 117.6°, at a density of 1.04 g/cm^3^ and the stacks running at 21° relative to the cylinder axis. This puts the transition dipoles for individual BChl at an angle of 55° with the cylinder axis (Fig. 5). For this tube model, xyz files are given in the Supplementary Information.

## Discussion

The 3D arrangement of BChls provides a strong scaffold, making the system both stable and flexible at the same time. For example, the tails can slide relative to each other between layers to establish different curvatures for building concentric tubes or curved lamellae in chlorosomes. For every packing mode, the modeling converged upon stable geometry-optimized structures and gave meaningful energy parameters.

The packing mode 1 has the lowest energy. As was shown by Li et al., differential interstack H-bonding between BChl *c* along parallel stacks with partial rotational averaging at ambient temperature can be accommodated in packing mode 1 concentric cylindrical packings, and the presence of two spectral components in the NMR data can be attributed to H-bond donor and non-donor BChl species (Li et al. 2018, 2020; Eric et al. 2023b).

The antiparallel dimer packing is only 0.3 eV higher in energy than the packing of the packing mode 1 (Supplementary information). The optical polarization data collected from single chlorosomes are thought to support a supra-helical symmetry with distinct optical transitions showing polarization parallel or perpendicular to the tube axis (Günther et al. 2016). Antiparallel dimers are generally not considered to lead to a valid packing mode for chlorosomes. The antiparallel placement of transition dipoles partially quenches the dipole moment. It would not support the formation of extended exitonic states in line with the cylindrical structure presented in Fig. 5 and would lead to a hypsochromic *vs*. bathochromic shift. It is, however, conceivable that a small fraction of stacks running antiparallel can destabilize to form stacks of dimers to explain the − 6 ppm cross-peak in the MAS NMR Hetcor spectrum, assigned to C-18^1^b. Ring current shifts close to the center of a single chlorophyll can be up to 7.2 ppm (Giessner-Prettre and Pullman 1971) For stacked chlorophylls ring current shifts are generated from both neighboring BChls in the stack and can be large (see e.g., Supplements of (Ganapathy et al. 2009)).

## Conclusions and implications

A specific packing model is constructed for chlorosomes from *C. tepidum* WT that is in agreement with NMR and cryo-EM data, and consistent with optical data. We analyzed different levels of heterogeneity within BChl *c* containing *syn-anti* motifs, compositional and dynamic. While chlorosomes are only weakly ordered, there exists a common pseudo-symmetric motif, which can be resolved by MAS NMR. By forming stacks and multiple layers concentric tubes are constructed, that can adapt to compositional heterogeneity and mutations. The packing shows little stereoselectivity for carbon C-3^1^ and is robust with respect to variations in the side chains.

With access to models that resolve the short and medium-range order in heterogeneous chlorosomes with compositional variability, a genuine understanding of how structure and dynamics in chlorosomes lead to their unique long-range transfer characteristics across different genotypes will be within reach. These principles may well be transferred to practical applications for building highly efficient artificial photoactive devices for light-harvesting and charge separation in solar fuel cells.

### Supplementary Information

Below is the link to the electronic supplementary material.

Supplementary material 1 (DOCX 3248.0 kb)
