# Supplementary material for: Contrasting packing modes for tubular assemblies in chlorosomes
Source: Photosynth Res. 2024 Mar 27;161(1-2):105–15. doi: 10.1007/s11120-024-01089-3 (PMC11269348; doi:10.1007/s11120-024-01089-3)
Supplement: Supplementary file 1 — Supplementary material 1 (DOCX 3248.0 kb) [file 11120_2024_1089_MOESM1_ESM.docx]

Supplementary Information

**Table of Contents**

[Absorbance of chlorosomes 2](#_Toc155720253)

[Pulse sequences used in the present study 3](#_Toc155720254)

[Assignment of ^13^C and ^1^H chemical shifts for chlorosomes from WT Cba. tepidum 4](#_Toc155720255)

[Construction of the repeat unit 1 for packing mode 1 6](#_Toc155720256)

[Simulation of Electron Microscopy 6](#_Toc155720257)

[Enantiomers of the packing mode 1 7](#_Toc155720258)

[Structure of alternating syn and anti stacks 8](#_Toc155720259)

[Hydrogen bonds 8](#_Toc155720260)

[Antiparallel dimer fraction 9](#_Toc155720261)

[Chemical shift calculations 10](#_Toc155720262)

[Stabilization of the BChl c aggregate 14](#_Toc155720263)

[HCH spectra 15](#_Toc155720264)

[References 16](#_Toc155720265)

[Author Contributions 16](#_Toc155720266)

Absorbance of chlorosomes


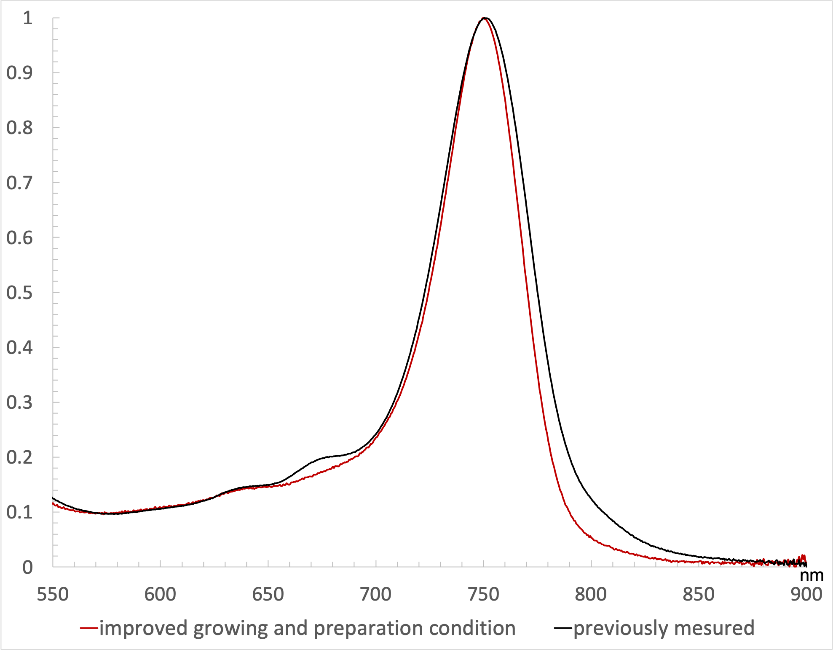
Figure S1 presents the absorbances of chlorosomes isolated from green sulphur bacterium *Cba. tepidum* measured for the current paper (red), and by (Ganapathy et al. 2009a) (black). For the current paper’s sample the growth was done at 125 μE for 1 day and the preparation used a milder purification method with NaSCN. For the sample from (Ganapathy et al. 2009a) the growth was done at 25 μE for 3 days and used a harsher detergent LDAO at purification step. The fact that our spectrum is narrower is the result of a combination of 3 effects: higher light intensity, milder purification method and a shorter growth length allowing for more homogenous size of the chlorosomes.

**Fig. S1** Absorbance of chlorosomes measured for this study (red) and for (Ganapathy et al. 2009a)(black). This illustrates the improved homogeneity for the sample preparation procedure developed by (Tian et al. 2011), compared to the sample preparation used in the earlier work. The traces are normalized with respect to the maximum.

Pulse sequences used in the present study


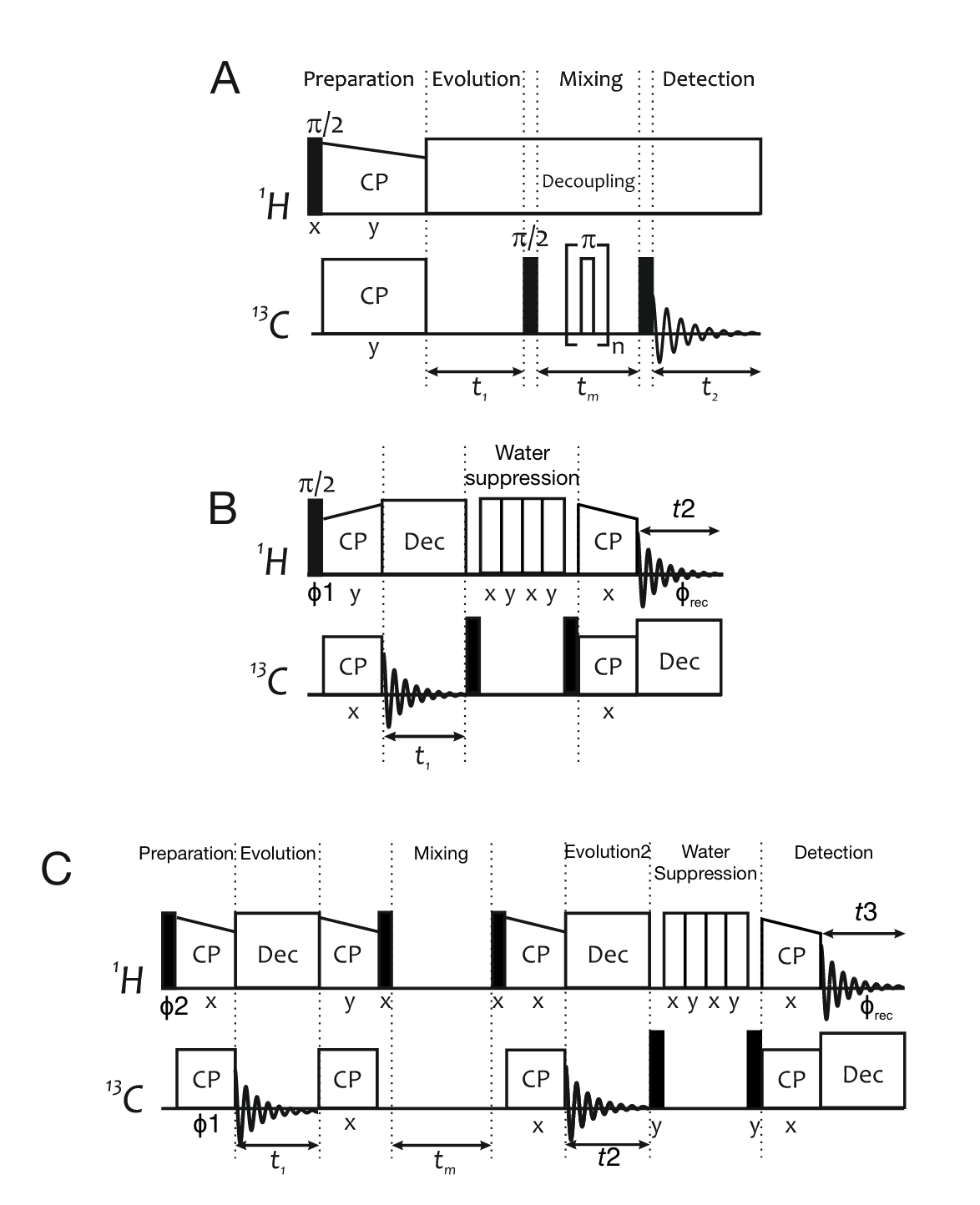


**Fig. S2** MAS NMR pulse sequences performed in the experiments. A) 2D ^13^C−^13^C homonuclear radiofrequency-driven dipolar recoupling (RFDR) for ^13^C chemical shifts assignments, B) 2D ^1^H−^13^C HCH for ^1^H chemical shifts assignments, C) 3D ^13^C−^13^C hChhCH for intermolecular correlations: φ_1_ = x -x, φ_2_ = x x -y -y, φ_rec_ = x -x -x x

Assignment of ^13^C and ^1^H chemical shifts for chlorosomes from *WT Cba. tepidum*

**Table S1** ^13^C chemical shifts for chlorosomes and BChl *c* aggregates from WT *Cba. tepidum* in ppm: liquid $\sigma_{liq}^{C}$, solid-state $\sigma_{s}^{C}$ and aggregation shifts $\Delta\sigma_{s}^{C}=\sigma_{s}^{C}-\sigma_{liq}^{C}$

| **Position** | $\boldsymbol{\sigma}_{\boldsymbol{liq}}^{\boldsymbol{C}}$,(**Balaban et al. 1995)** | $\boldsymbol{\sigma}_{\boldsymbol{s}}^{\boldsymbol{C}}$ **BChl *c* aggregates** | $\boldsymbol{\Delta}\boldsymbol{\sigma}_{\boldsymbol{s}}^{\boldsymbol{C}}$ **BChl *c* aggregates** | $\boldsymbol{\sigma}_{\boldsymbol{s}}^{\boldsymbol{C}}$ **chlorosomes** | $\boldsymbol{\Delta}\boldsymbol{\sigma}_{\boldsymbol{s}}^{\boldsymbol{C}}$ **chlorosomes** |
| --- | --- | --- | --- | --- | --- |
| **1a - C** | 153.8 | 153.5 | -0.3 | 154.2 | 0.4 |
| **1b - C** |  | 152.9 | -0.9 | - | - |
| **2a - C** | 135.2 | 134.4 | -0.8 | 134.8 | -0.4 |
| **2b - C** |  | 136.3 | 1.1 | - | - |
| **2^1^a - CH_3_** | 17 | 14.3 | -2.7 | 13.3 | -3.7 |
| **2^1^b - CH_3_** |  | 13.5 | -3.5 | - | - |
| **3 - C** | 145.2 | 139.0 | -6.2 | 139.4 | -5.8 |
| **3^1^a - CH** | 64.1 | 63.2 | -0.9 | 63 | -1.1 |
| **3^1^b - CH** |  | 62.7 | -1.4 | - | - |
| **3^2^a - CH_3_** | 25.6 | 22.0 | -3.6 | 23.3 | -2.3 |
| **3^2^b - CH_3_** |  | 24.3 | -1.3 | 20.8 | -4.8 |
| **4a - C** | 145.3 | 144.3 | -1.0 | 144.4 | -0.9 |
| **4b - C** |  | 143.0 | -2.3 | 143.5 | -1.8 |
| **5a - CH** | 100.2 | 94.6 | -5.6 | 94.5 | -5.7 |
| **5b - CH** |  | 100.9 | 0.7 | 100.7 | 0.5 |
| **6a - C** | 150.7 | 150.3 | -0.4 | 150.3 | -0.4 |
| **6b - C** |  | 149.4 | -1.3 | 149.2 | -1.5 |
| **7a - C** | 133.6 | 130.9 | -2.7 | 130.6 | -3 |
| **7b - C** |  | 132.3 | -1.3 | 132.1 | -1.5 |
| **7^1^a - CH_3_** | 10.6 | 6.6 | -4.0 | 6.5 | -4.1 |
| **7^1^b - CH_3_** |  | 10.4 | -0.2 | 10.1 | -0.5 |
| **8a - C** | 143.5 | 140.1 | -3.4 | - | - |
| **8b - C** |  | 141.9 | -1.6 | 141.8 | -1.7 |
| **8^1^ - CH_2_** | 19.2 | 17.8 | -1.4 | 18.5 | -0.7 |
| **8^2^ - CH_3_** | 17.1 | 18.2 | 1.1 | 16.9 | -0.2 |
| **9a - C** | 146.1 | 146.3 | 0.2 | 146.2 | 0.1 |
| **9b** |  | 145.8 | -0.3 | - | - |
| **10 - CH** | 105.6 | 104.8 | -0.8 | 104.7 | -0.9 |
| **11 - C** | 147.7 | 146.4 | -1.3 | 147.3 | -0.4 |
| **12 - C** | 140.7 | 138.5 | -2.2 | 138.7 | -2 |
| **12^1^- CH_2_** | 20.8 | 18.3 | -2.5 | 18.7 | -2.1 |
| **12^2^ - CH_3_** | 16.6 | 17.5 | 0.9 | 17.8 | 1.2 |
| **13 - C** | 131 | 127.1 | -3.9 | 126.7 | -4.3 |
| **13^1^ - C** | 198.2 | 195.8 | -2.4 | 196.3 | -1.9 |
| **13^2^ - CH_2_** | 49.1 | 47.7 | -1.4 | 48.1 | -1 |
| **14 - C** | 161.1 | 161.8 | 0.7 | 162.8 | 1.7 |
| **15 - C** | 104.9 | 103.7 | -1.2 | 103.5 | -1.4 |
| **16 - C** | 154.3 | 153.9 | -0.5 | 154.6 | 0.3 |
| **17 - CH** | 50.1 | 49.5 | -0.6 | 49.5 | -0.6 |
| **17^1^ - CH_2_** | 29.7 | 28.4 | -1.3 | 28.2 | -1.5 |
| **17^2^ - CH_2_** | 30.7 | 30.3 | -0.4 | 30.6 | -0.1 |
| **17^3^a - C** | 173.7 | 172.7 | -1.0 | 172.1 | -1.6 |
| **17^3^b - C** |  | 179.9 | 6.2 | - | - |
| **18a - CH** | 48.2 | 48.2 | 0.0 | 47.7 | -0.5 |
| **18b - CH** | 48.2 | 44.7 | -3.5 | 45 | -3.2 |
| **18^1^a - CH_3_** | 20.6 | 19.5 | -1.1 | 19.3 | 1 |
| **18^1^b - CH_3_** | 20.6 | 15.4 | -5.2 | 15.5 | -5.1 |
| **19a - C** | 167.8 | 167.0 | -0.8 | 168.8 | 1 |
| **19b - C** | 167.8 | 166.1 | -1.7 | 167.1 | -0.7 |
| **20 - C** | 104.7 | 103.8 | -0.9 | 104.1 | -0.6 |
| **20^1^ - CH_3_** | 21.1 | 20.0 | -1.1 | 19.8 | -1.3 |
| **F1 - CH_2_** | 61.3 | 60.6 | -0.7 | 59.6 | -1.7 |
| **F2 - CH** | 117.5 | 118.8 | 1.3 | 119.1 | 1.6 |
| **F3 - C** | 142.4 | 140.2 | -2.2 | 140.1 | -2.3 |
| **F3^1^ - CH_3_** | 16 | 14.9 | -1.1 | 15.4 | -0.6 |
| **F4 - CH_2_** | 39.3 | 39.1 | -0.2 | 38.7 | -0.6 |
| **F5 - CH_2_** | 26.4 | 26.0 | -0.4 | 25.2 | -1.2 |
| **F6 - CH** | 123.3 | 123.1 | -0.2 | 123.4 | 0.1 |
| **F7 - C** | 135.1 | 135.8 | 0.7 | 133.9 | -1.2 |
| **F7^1^ - CH_3_** | 15.5 | 14.6 | -0.9 | 12.2 | -3.3 |
| **F8 - CH_2_** | 39.1 | 39.1 | 0.0 | 38.9 | -0.2 |
| **F9 - CH_2_** | 25.8 | 25.0 | -0.8 | 24.6 | -1.2 |
| **F10 - CH** | 124 | 123.3 | -0.7 | 124.4 | 0.4 |
| **F11 - C** | 131 | 129.5 | -1.5 | 130.3 | -0.7 |
| **F11^1^ - CH_3_** | 17.2 | 16.8 | -0.4 | - | - |
| **F12 - CH_3_** | 25.2 | 24.6 | -0.6 | - | - |

**Table S2.** ^1^H chemical shifts for chlorosomes from WT *Cba. tepidum* in ppm: liquid $\sigma_{liq}^{H}$, solid-state $\sigma_{s}^{H}$ and aggregation shifts $\Delta\sigma_{s}^{H}=\sigma_{s}^{H}-\sigma_{liq}^{H}$.

| **Position** | $\boldsymbol{\sigma}_{\boldsymbol{liq}}^{\boldsymbol{H}}$**(van Rossum et al. 2001)** | $\boldsymbol{\sigma}_{\boldsymbol{s}}^{\boldsymbol{H}}$ **BChl *c* aggregates** | $\boldsymbol{\Delta}\boldsymbol{\sigma}_{\boldsymbol{s}}^{\boldsymbol{H}}$ **BChl *c* aggregates** | $\boldsymbol{\sigma}_{\boldsymbol{s}}^{\boldsymbol{H}}$ **chlorosomes** | $\boldsymbol{\Delta}\boldsymbol{\sigma}_{\boldsymbol{s}}^{\boldsymbol{H}}$ **chlorosomes** |
| --- | --- | --- | --- | --- | --- |
| **2^1^a - H_3_** | 3.3 | 0.4 | -2.9 | 0.7 | -2.6 |
| **2^1^b - H_3_** |  | 0.4 | -2.9 |  |  |
| **3^1^a - H** | 6.25 | 2.6 | -3.6 | 2.9 | -3.4 |
| **3^1^b - H** |  | 2.9 | -3.3 |  |  |
| **3^2^a - H_3_** | 2.01 | 0.1 | -1.9 | -0.1 | -2.1 |
| **3^2^b - H_3_** |  | 0.0 | -2.0 |  |  |
| **5a - H** | 9.56 | 6.9 | -2.7 | 7.1 | -2.5 |
| **5b - H** | 9.56 | 6.3 | -3.3 | 6.2 | -3.4 |
| **7^1^a - H_3_** | 3.19 | -1.2 | -4.4 | -1.1 | -4.3 |
| **7^1^b - H_3_** | 3.19 | 2.4 | -0.8 | 2.4 | -0.8 |
| **8^1^ - H_2_** | 3.67 | 1.1 | -2.6 | 2 | -1.7 |
| **8^2^ - H_3_** | 1.62 | 0.8 | -0.8 | 1.4 | -0.3 |
| **10 - H** | 9.43 | 8.8 | -0.7 | 8.8 | -0.9 |
| **12^1^ - H_2_** | 3.97 | 1.0 | -3.0 | 1.6 | -2.4 |
| **12^2^ - H_3_** | 1.79 | 0.1 | -1.7 |  |  |
| **13^2^ - H_2_** | 5.09 | 3.8 | -1.3 | 4 | -1.1 |
| **17 - H** | 4.08 | 3.4 | -0.6 | 3.6 | -0.5 |
| **17^1^ - H_2_** | 2.00/2.20 | 1.8 | -0.2 | 1.7 | -0.3 |
| **17^2^ - H_2_** | 2.35/2.42 | 2.1 | -0.2 | 1.8 | -0.6 |
| **18a - H** | 4.52 | 3.5 | -1.0 | 2.3 | -2.2 |
| **18b - H** | 4.52 | - | - | 2 | -2.5 |
| **18^1^a - H_3_** | 1.43 | 0.7 | -0.8 | 1 | -0.4 |
| **18^1^b - H_3_** | 1.43 | -6.2 | -7.7 | -6 | -7.4 |
| **20^1^ - H_3_** | 3.72 | 2.3 | -1.4 | 2.6 | -1.1 |
| **F1 - H_2_** | 4.31 | 4.0 | -0.3 | 4.2 | -0.1 |
| **F2 - H** | 5.05 | 4.2 | -0.8 | 4.9 | -0.1 |
| **F4 - H_2_** | 1.88 | 1.3 | -0.6 | 1.6 | -0.3 |
| **F5 - H_2_** | 1.88 | 1.7 | -0.2 | 1.9* | 0 |
| **F6 - H** | 4.95 | 2.9 | -2.1 | 4.5 | -0.4 |
| **F8 - H_2_** | 1.88 | - | - | 2 | 0.1 |
| **F9 - H_2_** | 1.88 | - | - | 1.4 | -0.4 |
| **F10 - H** | 4.95 | - | - | 5.2 | 0.3 |
| **F11^1^ - H_3_** | 1.5 | - | - | 1.5* | 0 |
| **F12 - H_3_** | 1.54 | 3.6 | 2.1 | 2.4* | 0.9 |

Construction of the repeat unit 1 for packing mode 1

To build the first packing mode 1, a *syn-anti* pair of [8-Me,12-Me]BChl *d* molecules was extracted from the *bchQRU* cylindrical model and converted into [8-Et,12-Et]BChl *c*, which is the most abundant homologue for the WT (Balaban et al. 1995; Ganapathy et al. 2009a). The tail was folded towards the ring, first in the direction of the Mg^2+^ doming and then to the other side of the macrocycle. This conformation is similar to the ethyl side chain in the X-ray structure of ethyl chlorophyllide *a* (Chow et al. 1975). The repeat unit 1 was optimized with the Dreiding molecular mechanics force field. A large starting separation of ~ 40 Å between layers was used, and it was found that the folding of the tails was stable.

Subsequently, the distance between the layers was decreased in steps to the 20-21 Å separation, measured with EM for the WT chlorosomes (Ganapathy et al. 2009a), while optimization was performed at each step. The optimized repeat unit 1 had the ends of the tails positioned in cavities in the planes, formed by the stacked macrocycles of an adjacent layer, to minimize packing constraints. In this way, a P1 repeat unit 1 in a periodic lattice with Pc pseudo-symmetry along the *syn-anti* stacks was obtained with fixed lattice parameters *a* = 12.9 Å, *b* = 9.8 Å, *c* = 21.5 Å and angles 90°, 90°, 122°. To refine the atomic positions in the repeat unit further, the strain on the model was released and all the lengths and angles of the repeat unit 1 were optimized additionally to the standard geometry optimization, first quickly by Dreiding force field, leading to the repeat unit with *a* = 14.1 Å, *b* = 10.1 Å, *c* = 20.9 Å and with angles 84°, 87°, 121°; and further with more elaborative first-principles quantum mechanics optimization, resulting in parameters: *a* = 14.7 Å, *b* = 9.8 Å, *c* = 22.7 Å and angles 82°, 85°, 118°. This extended the *a*-axis, while the angles and the *b*-axis did not change much during optimization. The C-12^2^ was rotated along the 12-12^1^ bond to achieve pseudosymmetric *syn* and *anti* BChls, which was validated with very similar ^1^H shifts, calculated with CASTEP (Tables S7 and S9). As a benchmark for the density, we used ethyl-chlorophyllide *a* dehydrate, for which a crystal structure is known and has ρ=1.31 g/cm^3^ and no tails (Chow et al. 1975). The density of our relaxed staking mode 1 model in a plain, with tails, is ρ ~ 0.95 g/cm^3^. This appears too low for an organic material, and the modelling strongly suggests that the repeat unit is compressible along the a-axis. When the density is calculated relative to the macromolecular ring after molecular dynamics optimization, it becomes 1.35 g/cm^3^ (Ganapathy et al. 2012). Curving the packing modes models into tubes leads to higher densities (Gomez Maqueo Chew et al. 2007; Li et al. 2018). These interleaved molecules also help to increase the density and stabilize the packing. Moreover, a chlorosome is estimated to contain 20-30% carotenoids, quinones and nonpolar lipids, that occupy open space between the tails (Adams et al. 2013).

To construct the tube model, shown in Fig. 5, the c-axis was extended to 42.7 Å to give the space between the layers/ surfaces, the boundary conditions were removed, and the adapted repeat unit was multiplied thirteen times in the *b* direction to produce a *b** = (13, -1, 0) rolling vector (surface) of length 121.3 Å at an angle of - 5.7° with the *b*-axis. Seamless tubes were then formed at different bending radii, corresponding with multiples of *b**/2π. Four sheets were generated and bent into cylinders with radii 38.6, 57.9, 77.2 and 96.5 Å, thus establishing the concentric tube model for the chlorosomes, that matches both the chlorosome EM layer line at 1/12.5 Å^-1^ and the perpendicular equatorial reflections at 1/21 Å^-1^ (Fig. S4).

Simulation of Electron Microscopy

Figure S4 shows the EM projection of four cylinders with radii 38.6, 57.9, 77.2 and 96.5 Å with BChls packed in packing mode 1, which match the experimental EM spectrum from (Oostergetel et al. 2007).

**Fig. S4** (Left Panel) Simulated EM image from a tubular aggregate built from four concentric tubes, made by wrapping sheets of packing mode 1 onto cylinders along the rolling vector in Fig. 5. The right panel shows the Fourier transform (FT) with the strong equatorial reflections at 1/21 Å^-1^ and the meridional layer line reflections at 1/12.5 Å^-1^, in line with the cryo-EM data for the WT (Oostergetel et al. 2007).

Enantiomers of the packing mode 1

BChls with *R* and *S* stereochemistries at C-3^1^ were tested in order to identify energetically stable packings. Four pairs of BChls were analysed: with the same chirality, *RR* or *SS*, and with alternating, *SR* or *RS*. The results obtained with the local orbital DMol^3^ DFT method for fixed repeat unit 1, shown in Table S3, confirm that there is no any preference for particular enantiomer organization at C-3^1^.

Also, various BChl *c* homologues with ethyl (Et), or propyl (Pr) substituents at positions C-8 and C-12 were investigated for their influence on the packing. The BChl *c* with C-8 Et and C-12 Et was found to constitute the largest fraction (~55%) among the homologs, with the second most abundant being BChl *c* with C-8 Et and C-12 Pr (~38%), which can self-aggregate from hexane to form a structure with virtually the same NMR response as for the natural system (Balaban et al. 1995).

Since various homologs with different numbers of atoms were optimized, because of different substituents at C-8 and C-12, the energy parameters were normalized by the number of atoms. The differences in total energy for *syn-anti* combinations of epimers are up to ~ 100 meV, comparable to what was calculated with the CASTEP method, which validated the local orbital approach (not shown). The models with the same number of atoms had the same normalized total energy without any preference for particular enantiomer organization at C-3^1^. The higher the number of atoms, the higher was the total energy and the sum of atomic energies and the lower was the binding energy, which is the energy needed to dissociate the crystal into atoms at infinite separation. This suggests some destabilization from the larger substituents by steric hindrance when the parameters of repeat units are kept the same. Considering that the variation of the repeat unit was minimal for different mixtures in the modelling as well, we took the repeat unit 1 with [C-8 Et, C-12 Et] BChl *c* with *syn S* and *anti R* epimer units as the epitome for the chlorosome structure.

**Table S3** Energy components contributions from the DMol^3^ geometry optimization on the fixed repeat unit 1 with the most abundant homologs. Fixed means that the parameters *a*, *b*, *c* and angles between them were kept fixed and not changed during this particular calculation.

| **BChl *c* homologues** | **[8-Et,12-Et]** | | | | **[8-Pr,12-Et]** | | | |
| --- | --- | --- | --- | --- | --- | --- | --- | --- |
|  | *RS* | *SR* | *SS* | *RR* | *RS* | *SR* | *SS* | *RR* |
| **Sum of atomic energies, keV** | -143.0 | -143.0 | -143.0 | -143.0 | -145.1 | -145.1 | -145.1 | -145.1 |
| **Total Energy, keV** | -144.2 | -144.2 | -144.2 | -144.2 | -146.3 | -146.3 | -146.3 | -146.3 |
| **df binding energy, eV** | -1234.5 | -1234.5 | -1234.5 | -1234.3 | -1261.6 | -1261.5 | -1261.5 | -1261.4 |
| **number of atoms** | 242 | 242 | 242 | 242 | 248 | 248 | 248 | 248 |
| **Total Energy, eV/atom** | -595.9 | -595.9 | -595.9 | -595.9 | -590.1 | -590.1 | -590.1 | -590.1 |
| **binding energy, eV/atom** | -5.10 | -5.10 | -5.10 | -5.10 | -5.09 | -5.09 | -5.09 | -5.09 |

Structure of alternating *syn* and *anti* stacks

The parallel alternating *syn* and *anti* stacks, like repeat unit 2 (Fig. 1), was constructed on the basis of the repeat unit 1 while preserving H-bonding and the Mg-Mg distances between BChl *c* molecules. It was inspired by a bchQR mutant containing predominantly the (C-31-R)-[8-ethyl,12-methyl]BChl c homologue, that self-assembly into all-*syn* and all-*anti* stacks, in line with an alternating *syn* and *anti* stacks packing ([Ganapathy et al. 2012](#_ENREF_22)). Alternating *syn* and *anti* stacks differs from the packing mode 1 in that the stacks are going not along the *syn-ant-*BChl connections via Mg coordination, but perpendicular to that. Stacks consist of all-*syn* and all-*anti* BChls, which are connected via H-bond between the C=O and the 3C-OH oxygens at the distances of 1.8 and 2.7 Å. This is in the same range with packing mode 1.

**Fig. S5** Variability in chlorosome structure due to heterogeneity: alternating *syn* and *anti* parallel stacks. A. Schematic patterns of *syn* (purple) and *anti* (yellow) BChls. Green triangle marks the triangular lattice. B. View from the top of the stacks with marked hydrogen bonds (in cyan). Note the all-*syn-*all-*anti* stacks in alternating *syn* and *anti* stacks.

Hydrogen bonds

**Fig. S6** View from the top of the stacks with marked hydrogen bonds (in cyan). Both packing modes 1 and 2 can be stabilized by interstack H-bonding on both sides of the sheets. For the packing mode 1 a *syn-anti* pair in the middle is colored in purple and yellow. For the alternating *syn* and *anti* parallel stacks the hydrogen bonding alternates between the front and the back of the sheets.

Fig. S6 presents the top view on the packing modes, when looking through the stacks. Both packing modes 1 and 2 have well-ordered *syn*-*anti* stacks with connecting H-bonds (marked in cyan), forming a zig-zag pattern with two types of BChl orientations between two stacks: from *syn* to *anti* and from *anti* to *sin*. Alternating *syn* and *anti* stacks arrangement, however, has only one and the same type of a hydrogen bond between its two all-*syn* and all-*anti* stacks. This lack of zigzag pattern seems to bring a lot of freedom to its structure and less robustness against bending, allowing the alternating *syn* and *anti* stacks to more easily accommodate tubes to a smaller radius, by the rotation of entire stacks in the structure. Tails in the alternating *syn* and *anti* stacks are turned to one side of the stack, leaving another side empty. Optimized parameters of repeat units are listed in Table S4.

**Table S4** Repeat units used in CASTEP to generate periodic packing modes, after optimization. They are provided as .cif files

| **[8-Et,12-Et]-****BChl *c*** |  | **Length** | | | **Angle** | | |
| --- | --- | --- | --- | --- | --- | --- | --- |
|  | **Type** | **a** | **b** | **c** | ***α*** | ***β*** | ***γ*** |
|  | **Packing mode 1** | 14.66 | 9.77 | 22.71 | 82.30 | 84.82 | 117.62 |
|  | **Packing mode 2** | 15.00 | 21.67 | 20.64 | 95.94 | 87.19 | 153.61 |
|  | **Alternating *syn* and *anti* stacks** | 7.38 | 36.77 | 20.50 | 78.55 | 99.91 | 152.84 |
|  | **antiparallel dimer fraction** | 14.96 | 11.80 | 19.68 | 88.86 | 90.26 | 128.09 |
| **[8-Pr,12-Et]- BChl *c*** | **Packing mode 1** | 14.36 | 10.15 | 21.30 | 89.17 | 84.19 | 120.93 |

Antiparallel dimer fraction

A minor fraction, as observed by NMR, can possibly result from two *syn* and *anti* BChls forming a closed (antiparallel) dimer with Mg atoms coordinating OH-groups of each of the molecule, stabilized by intermolecular H-bonds. This gives a zig-zag type pattern without clear stacks. In addition, since dipoles are quenched in the dimer to a large extent, this cannot lead to the large extended dipoles that are required for harvesting light by chlorosomes.

Chemical shift calculations

Four packing modes were analysed with CASTEP to calculate ^13^C and ^1^H chemical shifts (Tables S5-8). The detailed procedure is given in the Modelling subsection of the Materials and methods section. Chemical shifts from both *syn*- and *anti-*BChls are listed. RMSD values were calculated from the deviations from experimental chemical shifts.

**Table S5** ^13^C chemical shifts for the atoms of the *syn-* and *anti*-BChls *c* in packing modes 1 and 2 calculated by CASTEP. Deviations with the experimental data are calculated as *σ_s_^C^_expt_* - *σ_s_^C^_calc_*. Deviations between the calculated chemical shifts of *syn-* and *anti-*BChls are *σ_s_^C^_cal_* (*syn*)- *σ_s_^C^_cal_* (*anti*)

| **Position** | **Data^[a]^** | **Calculated** **for packing mode 1** | | | | | **Calculated for packing mode 2** | | | | |  |
| --- | --- | --- | --- | --- | --- | --- | --- | --- | --- | --- | --- | --- |
|  | ***σ_s_^C^_exp_***  **BChl** | ***σ_s_^C^_cal_* (*syn*)**  **^[b]^** | ***σ_s_^C^_expt_*- *σ_s_^C^* *_cal_* (*syn*)** | ***σ_s_^C^_cal_* (*anti*)^[b]^** | ***σ_s_^C^_expt_*- *σ_s_^C^_cal_* (*anti*)** | ***σ_s_^C^_cal_* (*syn*)- *σ_s_^C^_cal_* (*anti*)** | ***σ_s_^C^_cal_* (*syn*)^[b]^** | ***σ_s_^C^_expt_*- *σ_s_^C^* *_cal_* (*syn*)** | ***σ_s_^C^_cal_* (*anti*)^[b]^** | ***σ_s_^C^_expt_*- *σ_s_^C^* *_cal_* (*anti*)** | ***σ_s_^C^_cal_* (*syn*)- *σ_s_^C^_cal_* (*anti*)** | |
| **1b - C** | 152.9 | 147.7 | 5.2 | 144.2 | 8.7 | 3.5 | 139.6 | 13.3 | 141.3 | 11.6 | -1.6 | |
| **2b - C** | 136.3 | 133.7 | 2.6 | 137.6 | -1.4 | -4.0 | 136.1 | -1.7 | 132.4 | 2.0 | 3.7 | |
| **2^1^b - CH_3_** | 13.2 | 3.7 | 9.5 | 4.7 | 8.5 | -1.0 | 4.8 | 8.4 | 6.0 | 7.2 | -1.2 | |
| **3 - C** | 139.0 | 128.9 | 10.1 | 133.2 | 5.8 | -4.3 | 128.6 | 10.4 | 134.6 | 4.4 | -6.0 | |
| **3^1^a - CH** | 63.0 | 66.0 | -2.9 | 65.3 | -2.2 | 0.7 | 64.1 | -1.1 | 65.0 | -1.9 | -0.9 | |
| **3^2^b - CH_3_** | 24.2 | 23.3 | 0.9 | 23.8 | 0.4 | -0.5 | 28.2 | -4.1 | 29.7 | -5.5 | -1.4 | |
| **4b - C** | 143.0 | 135.5 | 7.5 | 134.5 | 8.5 | 1.0 | 135.8 | 7.1 | 133.7 | 9.2 | 2.1 | |
| **5a - CH** | 94.5 | 93.1 | 1.4 | 93.8 | 0.7 | -0.7 | 96.6 | 4.2 | 143.7 | -42.9 | -47.1 | |
| **6b - C** | 149.2 | 143.7 | 5.6 | 142.7 | 6.5 | 0.9 | 141.9 | 7.4 | 140.5 | 8.7 | 1.4 | |
| **7b - C** | 132.1 | 134.4 | -2.3 | 136.9 | -4.8 | -2.5 | 133.7 | -1.6 | 135.8 | -3.7 | -2.1 | |
| **7^1^a - CH_3_** | 6.5 | 4.8 | 1.7 | 4.5 | 2.0 | 0.3 | 3.1 | 3.4 | 3.3 | 3.2 | -0.2 | |
| **8b - C** | 141.9 | 145.6 | -3.8 | 145.6 | -3.7 | 0.1 | 144.8 | -2.9 | 147.7 | -5.9 | -3.0 | |
| **8^1^ - CH_2_** | 17.9 | 18.4 | -0.4 | 18.4 | -0.5 | 0.0 | 17.7 | 0.3 | 18.6 | -0.6 | -0.9 | |
| **8^2^ - CH_3_** | 17.5 | 14.4 | 3.1 | 12.2 | 5.3 | 2.2 | 15.4 | 2.1 | 15.8 | 1.6 | -0.5 | |
| **9b - C** | 145.7 | 142.1 | 3.6 | 142.0 | 3.7 | 0.1 | 140.0 | 5.7 | 138.4 | 7.3 | 1.6 | |
| **10 - CH** | 104.7 | 100.4 | 4.3 | 101.3 | 3.4 | -0.9 | 99.3 | 5.3 | 96.6 | 8.0 | 2.7 | |
| **11 - C** | 146.3 | 143.9 | 2.4 | 144.4 | 1.9 | -0.5 | 143.6 | 2.7 | 140.3 | 6.0 | 3.3 | |
| **12 - C** | 138.2 | 140.4 | -2.1 | 142.1 | -3.8 | -1.7 | 145.4 | -7.2 | 141.1 | -2.9 | 4.3 | |
| **12^1^- CH_2_** | 18.2 | 14.9 | 3.3 | 14.3 | 3.9 | 0.5 | 15.4 | 2.8 | 16.1 | 2.1 | -0.7 | |
| **12^2^ - CH_3_** | 17.5 | 12.0 | 5.5 | 10.0 | 7.5 | 2.0 | 10.2 | 7.3 | 6.3 | 11.1 | 3.9 | |
| **13 - C** | 126.8 | 120.2 | 6.6 | 123.7 | 3.1 | -3.5 | 122.0 | 4.8 | 122.2 | 4.6 | -0.3 | |
| **13^1^ - C** | 197.5 | 196.0 | 1.5 | 198.9 | -1.3 | -2.9 | 201.0 | -3.5 | 203.7 | -6.2 | -2.7 | |
| **13^2^ - CH_2_** | 47.6 | 43.8 | 3.8 | 41.9 | 5.7 | 1.9 | 42.7 | 4.9 | 41.5 | 6.1 | 1.2 | |
| **14 - C** | 161.7 | 152.6 | 9.1 | 152.7 | 9.0 | -0.1 | 151.9 | 9.9 | 149.2 | 12.5 | 2.7 | |
| **15 - C** | 103.6 | 112.0 | -8.3 | 108.5 | -4.8 | 3.5 | 108.4 | -4.7 | 107.8 | -4.1 | 0.6 | |
| **16 - C** | 153.8 | 154.7 | -0.9 | 159.0 | -5.2 | -4.3 | 152.8 | 1.0 | 157.4 | -3.6 | -4.6 | |
| **17 - CH** | 49.3 | 49.5 | -0.2 | 52.8 | -3.5 | -3.3 | 51.9 | -2.6 | 53.1 | -3.8 | -1.2 | |
| **17^1^ - CH_2_** | 30.2 | 37.1 | -6.9 | 34.5 | -4.3 | 2.6 | 42.2 | -12.0 | 30.7 | -0.5 | 11.5 | |
| **17^2^ - CH_2_** | 28.2 | 24.2 | 4.0 | 30.3 | -2.1 | -6.1 | 26.4 | 1.8 | 22.7 | 5.5 | 3.6 | |
| **17^3^b - CO** | 179.9 | 181.2 | -1.2 | 183.6 | -3.7 | -2.4 | 183.5 | -3.5 | 182.9 | -3.0 | 0.5 | |
| **18a - CH** | 48.1 | 48.9 | -0.8 | 50.4 | -2.3 | -1.5 | 43.5 | 1.5 | 47.8 | -2.8 | -4.3 | |
| **18^1^b - CH_3_** | 15.4 | 15.1 | 0.3 | 13.7 | 1.7 | 1.5 | 20.9 | -5.5 | 10.9 | 4.5 | 10.1 | |
| **19a - C** | 167.3 | 171.7 | -4.4 | 164.6 | 2.7 | 7.1 | 166.9 | -1.0 | 152.1 | 13.8 | 14.8 | |
| **20 - C** | 103.7 | 106.5 | -2.7 | 109.6 | -5.8 | -3.1 | 104.4 | -0.7 | 101.6 | 2.1 | 2.8 | |
| **20^1^ - CH_3_** | 19.9 | 15.1 | 4.7 | 15.7 | 4.2 | -0.5 | 20.4 | -0.5 | 14.0 | 5.9 | 6.4 | |
| **F1 - CH_2_** | 60.6 | 69.7 | -9.2 | 61.3 | -0.7 | 8.4 | 56.4 | 4.2 | 61.9 | -1.3 | -5.4 | |
| **F2 - CH** | 118.8 | 123.4 | -4.6 | 119.2 | -0.5 | 4.1 | 116.4 | 2.4 | 110.8 | 8.0 | 5.6 | |
| **F3 - C** | 140.2 | 139.3 | 0.9 | 151.6 | -11.5 | -12.4 | 152.5 | -12.3 | 162.3 | -22.1 | -9.8 | |
| **F3^1^ - CH_3_** | 14.9 | 15.5 | -0.7 | 10.9 | 4.0 | 4.6 | 13.0 | 1.9 | 16.5 | -1.6 | -3.5 | |
| **F4 - CH_2_** | 39.1 | 36.7 | 2.4 | 43.4 | -4.4 | -6.8 | 35.4 | 3.6 | 34.4 | 4.6 | 1.0 | |
| **F5 - CH_2_** | 26.0 | 30.5 | -4.5 | 28.5 | -2.5 | 2.1 | 25.3 | 0.7 | 28.8 | -2.8 | -3.6 | |
| **F6 - CH** | 123.1 | 124.8 | -1.6 | 119.9 | 3.2 | 4.8 | 124.5 | -1.3 | 128.8 | -5.6 | -4.3 | |
| **F7 - C** | 135.8 | 141.4 | -5.6 | 148.9 | -13.1 | -7.5 | 144.6 | -8.7 | 137.5 | -1.6 | 7.1 | |
| **F7^1^ - CH_3_** | 14.6 | 11.0 | 3.5 | 10.5 | 4.1 | 0.6 | 12.7 | 1.9 | 11.4 | 3.2 | 1.3 | |
| **F8 - CH_2_** | 39.1 | 42.1 | -3.0 | 39.3 | -0.2 | 2.8 | 40.4 | -1.3 | 40.4 | -1.3 | 0.0 | |
| **F9 - CH_2_** | 25.0 | 30.0 | -5.0 | 32.6 | -7.6 | -2.6 | 28.4 | -3.4 | 25.1 | -0.1 | 3.3 | |
| **F10 - CH** | 123.3 | 122.1 | 1.3 | 124.3 | -0.9 | -2.2 | 124.6 | -1.3 | 119.7 | 3.6 | 5.0 | |
| **F11 - C** | 129.5 | 134.8 | -5.2 | 133.3 | -3.8 | 1.4 | 128.9 | 0.6 | 137.6 | -8.1 | -8.7 | |
| **F11^1^ - CH_3_** | 16.8 | 11.0 | 5.8 | 14.1 | 2.7 | -3.1 | 11.9 | 4.9 | 14.8 | 1.9 | -3.0 | |
| **F12 - CH_3_** | 24.6 | 24.1 | 0.5 | 26.0 | -1.4 | -1.9 | 22.5 | 2.1 | 15.4 | 9.2 | 7.2 | |
| **RMSD** |  |  | 4.6 |  | 5.0 |  |  | 5.3 |  | 8.9 |  | |

[a] Experimental data from this work.

[b] Chemical shifts were referenced judging by the least deviation from the experimental data. The reference number for the *syn*-BChl in packing mode 1 was 169 and for the *anti*-BChl – 168. The reference for both BChls in packing mode 2 was 174. In case of doublings the experimental shift closest to the calculated was included in the table.

**Table S6** ^1^H chemical shifts for the atoms of the *syn* and *anti* BChls *c* in packing modes 1 and 2 calculated by CASTEP. Deviations with the experimental data are calculated as *σ_s_^H^_expt_* - *σ_s_^H^_calc_*. Deviations between the calculated chemical shifts of *syn* and *anti* BChls are *σ_s_^H^_cal_* (*syn*)- *σ_s_^H^_cal_* (*anti*)

| **Position** | **Data^[a]^** | **Calculated for packing mode 1** | | | | | **Calculated for packing mode 2** | | | | |
| --- | --- | --- | --- | --- | --- | --- | --- | --- | --- | --- | --- |
|  | ***σ_s_^H^_exp_***  **BChl** | $\boldsymbol{\sigma}_{\boldsymbol{s}}^{\boldsymbol{H}}$***_cal_* (*syn)*^[b]^** | ***σ_s_^H^_expt_*- *σ_s_^H^_cal_* (*syn*)** | $\boldsymbol{\sigma}_{\boldsymbol{s}}^{\boldsymbol{H}}$***_cal_***  **(*anti)*^[b]^** | ***σ_s_^H^_expt_*- *σ_s_^H^_cal_* (*anti*)** | ***σ_s_^H^_cal_* (*syn*)- *σ_s_^H^_cal_* (*anti*)** | ***σ_s_^H^_cal_***  ***(syn)*^[b]^** | ***σ_s_^H^_expt_* - *σ_s_^H^_cal_* (*syn*)** | ***σ_s_^H^_cal_***  ***(anti)*^[b]^** | ***σ_s_^H^_expt_* - *σ_s_^H^_cal_* (*anti*)** | ***σ_s_^H^_cal_* (*syn*)- *σ_s_^H^_cal_* (*anti*)** |
| **2^1^b - H_3_** | 0.2 | -2.0 | 2.1 | -2.2 | 2.3 | 0.2 | -1.5 | 1.7 | -2.1 | 2.2 | 0.6 |
| **3^1^a - H** | 2.6 | 0.9 | 1.7 | 1.6 | 1.0 | -0.7 | 0.2 | 2.4 | 1.0 | 1.6 | -0.8 |
| **3^2^b - H_3_** | 0.0 | -2.7 | 2.7 | -2.7 | 2.7 | 0.0 | -2.7 | 2.8 | -1.1 | 1.2 | -1.6 |
| **5b - H** | 6.3 | 4.7 | 1.5 | 5.1 | 1.2 | -0.3 | 4.3 | 2.0 | 4.9 | 1.4 | -0.5 |
| **7^1^b - H_3_** | 2.4 | 1.7 | 0.7 | 1.8 | 0.6 | -0.1 | 1.0 | 1.4 | 1.1 | 1.3 | 0.0 |
| **8^1^ - H_2_** | 2.0 | 2.4 | -0.3 | 2.4 | -0.4 | -0.1 | 1.5 | 0.5 | 1.3 | 0.7 | 0.2 |
| **8^2^ - H_3_** | 0.5 | 0.3 | 0.2 | 0.2 | 0.3 | 0.2 | -0.2 | 0.7 | -1.3 | 1.8 | 1.1 |
| **10 - H** | 8.8 | 4.7 | 4.1 | 5.0 | 3.8 | -0.3 | 4.2 | 4.6 | 3.4 | 5.4 | 0.9 |
| **12^1^ - H_2_** | 1.0 | -1.2 | 2.2 | -1.7 | 2.7 | 0.5 | -0.3 | 1.3 | -0.9 | 2.0 | 0.6 |
| **12^2^ - H_3_** | 0.1 | -6.5 | 6.5 | -6.3 | 6.3 | -0.2 | -6.6 | 6.7 | -7.0 | 7.1 | 0.4 |
| **13^2^ - H_2_** | 3.8 | 2.7 | 1.1 | 3.1 | 0.7 | -0.4 | 1.8 | 1.9 | 3.4 | 0.3 | -1.6 |
| **17 - H** | 3.4 | 3.2 | 0.2 | 2.9 | 0.6 | 0.4 | 1.8 | 1.6 | 2.9 | 0.5 | -1.1 |
| **17^1^ - H_2_** | 2.2 | 2.0 | 0.2 | 1.0 | 1.2 | 1.0 | 1.2 | 0.9 | 0.1 | 2.1 | 1.1 |
| **17^2^ - H_2_** | 2.0 | 2.3 | -0.3 | 2.0 | 0.0 | 0.3 | 1.5 | 0.5 | 1.1 | 0.9 | 0.4 |
| **18a - H** | 3.5 | 3.6 | -0.1 | 3.5 | 0.0 | 0.1 | 2.9 | 0.6 | 3.6 | -0.1 | -0.7 |
| **18^1^a - H_3_** | 0.7 | 0.6 | 0.1 | 0.4 | 0.2 | 0.1 | 0.2 | 0.5 | 0.1 | 0.6 | 0.1 |
| **20^1^ - H_3_** | 2.3 | 1.4 | 0.8 | 1.4 | 0.9 | 0.1 | 1.8 | 0.5 | -0.7 | 2.9 | 2.5 |
| **RMSD** |  |  | 2.2 |  | 2.5 |  |  | 2.4 |  | 2.6 |  |

[a] Experimental data from this work.

[b] Chemical shifts were referenced judging by the least deviation from the experimental data. The reference number for the both packing modes 1 and 2 was 29. In case of doublings the experimental shift closest to the calculated was included in the table.

**Table S7** ^13^C chemical shifts for the atoms of the *syn* and *anti* BChls *c* in alternating *syn* and *anti* stacks and antiparallel dimers, calculated by CASTEP. Deviations with the experimental data are calculated as *σ_s_^C^_expt_* - *σ_s_^C^_calc_*. Deviations between the calculated chemical shifts of *syn* and *anti* BChls are *σ_s_^C^_cal_* (*syn*)- *σ_s_^C^_cal_* (*anti*)

| \| **Position** \| \| --- \| \| | **Data^[a]^** | **Calculated for the** **alternating *syn* and *anti* stacks** | | | | | **Calculated for the antiparallel dimers** | | | | |
| --- | --- | --- | --- | --- | --- | --- | --- | --- | --- | --- | --- | --- |
|  | ***σ_s_^C^_expt_* BChl** | ***σ_s_^C^_cal_* (*syn*)^[b]^** | ***σ_s_^C^_expt_*- *σ_s_^C^* *_cal_* (*syn*)** | ***σ_s_^C^_cal_* (*anti*)^[b]^** | ***σ_s_^C^_expt_*- *σ_s_^C^_cal_* (*anti*)** | ***σ_s_^C^_cal_* (*syn*)- *σ_s_^C^_cal_* (*anti*)** | ***σ_s_^C^_cal_* (*syn*)**  **^[b]^** | ***σ_s_^C^_expt_*- *σ_s_^C^* *_cal_* (*syn*)** | ***σ_s_^C^_cal_* (*anti*)**  **^[b]^** | ***σ_s_^C^_expt_*- *σ_s_^C^_cal_* (*anti*)** | ***σ_s_^C^_cal_* (*syn*)- *σ_s_^C^_cal_* (*anti*)** |
| **1b - C** | 152.9 | 140.2 | 12.7 | 135.6 | 17.3 | 4.6 | 152.5 | 0.4 | 151.7 | 1.2 | 0.8 |
| **2a - C** | 134.4 | 122.2 | 12.1 | 129.7 | 4.6 | -7.5 | 138 | -1.7 | 140.4 | -4.1 | -2.4 |
| **2^1^b - CH_3_** | 13.2 | 6.1 | 7.1 | 7.2 | 6.0 | -1.1 | 4.9 | 8.3 | 9.8 | 3.4 | -4.9 |
| **3 - C** | 139.0 | 128.1 | 10.9 | 132.4 | 6.6 | -4.3 | 142.3 | -3.3 | 139.7 | -0.7 | 2.6 |
| **3^1^a - CH** | 63.0 | 66.7 | -3.6 | 64.4 | -1.3 | 2.3 | 66.1 | -3.1 | 65.5 | -2.5 | 0.6 |
| **3^2^b - CH_3_** | 24.2 | 20.3 | 1.3 | 19.6 | 2.0 | 0.8 | 16.5 | 5.1 | 23.6 | -2.0 | -7.1 |
| **4b - C** | 143.0 | 124.6 | 18.4 | 123.2 | 19.7 | 1.4 | 146.2 | -2.1 | 145.9 | -1.8 | 0.3 |
| **5b - CH** | 100.7 | 90.2 | 4.3 | 88.1 | 6.4 | 2.1 | 102.7 | -2.0 | 102.1 | -1.4 | 0.6 |
| **6b - C** | 149.2 | 139.0 | 10.2 | 134.1 | 15.1 | 4.9 | 150.9 | -0.6 | 153.3 | -3.0 | -2.4 |
| **7b - C** | 132.1 | 129.2 | 1.5 | 131.5 | -0.8 | -2.3 | 135.7 | -3.6 | 138 | -5.9 | -2.3 |
| **7^1^a - CH_3_** | 6.5 | 2.3 | 4.2 | 2.3 | 4.2 | 0.0 | 3.4 | 3.1 | -0.4 | 6.9 | 3.8 |
| **8b - C** | 141.9 | 141.3 | -1.6 | 139.7 | 0.0 | 1.6 | 142.7 | -0.8 | 146.5 | -4.6 | -3.8 |
| **8^1^ - CH_2_** | 17.9 | 13.0 | 5.0 | 14.2 | 3.8 | -1.2 | 14.4 | 3.5 | 13.3 | 4.6 | 1.1 |
| **8^2^ - CH_3_** | 17.5 | 11.3 | 6.2 | 11.8 | 5.7 | -0.5 | 7.1 | 11.1 | 6.3 | 11.9 | 0.8 |
| **9b - C** | 145.7 | 133.6 | 12.0 | 133.5 | 12.2 | 0.2 | 149.6 | -3.4 | 150.2 | -4.0 | -0.6 |
| **10 - CH** | 104.7 | 98.9 | 5.7 | 91.9 | 12.7 | 7.0 | 104 | 0.7 | 102.8 | 1.9 | 1.2 |
| **11 - C** | 146.3 | 141.2 | 5.1 | 141.6 | 4.7 | -0.4 | 150.3 | -4.0 | 149.3 | -3.0 | 1 |
| **12 - C** | 138.2 | 135.3 | 3.0 | 131.9 | 6.4 | 3.4 | 140.1 | -1.9 | 142.2 | -4.0 | -2.1 |
| **12^1^- CH_2_** | 18.2 | 13.0 | 5.2 | 10.6 | 7.6 | 2.3 | 17.1 | 1.1 | 17.4 | 0.8 | -0.3 |
| **12^2^ - CH_3_** | 17.5 | 6.0 | 11.5 | 3.7 | 13.7 | 2.3 | 7.7 | 9.8 | 8.6 | 8.9 | -0.9 |
| **13 - C** | 126.8 | 120.4 | 6.4 | 122.3 | 4.5 | -1.9 | 132.7 | -5.9 | 133.9 | -7.1 | -1.2 |
| **13^1^ - C** | 197.5 | 188.1 | 9.5 | 194.5 | 3.1 | -6.4 | 199.7 | -2.2 | 203.2 | -5.7 | -3.5 |
| **13^2^ - CH_2_** | 47.6 | 38.9 | 8.7 | 36.6 | 11.0 | 2.3 | 46.2 | 1.4 | 48.2 | -0.6 | -2 |
| **14 - C** | 161.7 | 148.4 | 13.3 | 145.4 | 16.3 | 3.0 | 166.8 | -5.1 | 164.4 | -2.7 | 2.4 |
| **15 - C** | 103.6 | 105.8 | -2.2 | 102.5 | 1.1 | 3.3 | 107.4 | -3.8 | 109.8 | -6.2 | -2.4 |
| **16 - C** | 153.8 | 149.0 | 4.8 | 156.3 | -2.5 | -7.3 | 155.9 | -2.1 | 157.7 | -3.9 | -1.8 |
| **17 - CH** | 49.3 | 46.9 | 2.4 | 52.8 | -3.5 | -5.9 | 49.9 | -0.6 | 46.8 | 2.5 | 3.1 |
| **17^1^ - CH_2_** | 30.2 | 20.8 | 9.4 | 36.0 | -5.8 | -15.2 | 12 | 18.2 | 13.4 | 16.8 | -1.4 |
| **17^2^ - CH_2_** | 28.2 | 29.9 | -1.7 | 27.3 | 0.9 | 2.6 | 22.8 | 5.4 | 18.4 | 9.8 | 4.4 |
| **17^3^b - CO** | 179.9 | 179.2 | 0.8 | 182.7 | -2.7 | -3.5 | 178.9 | 1.0 | 177.7 | 2.2 | 1.2 |
| **18b - CH** | 45.0 | 48.0 | 0.0 | 46.8 | 1.3 | 1.2 | 46 | -1.0 | 44.9 | 0.1 | 1.1 |
| **18^1^b - CH_3_** | 15.4 | 12.2 | 3.2 | 15.1 | 0.3 | -3.0 | -0.5 | 15.9 | 9.5 | 5.9 | -10 |
| **19b - C** | 165.9 | 140.2 | 25.7 | 158.6 | 7.3 | -18.4 | 168.6 | -1.3 | 168 | -0.7 | 0.6 |
| **20 - C** | 103.7 | 122.2 | -18.5 | 100.6 | 3.2 | 21.7 | 108.1 | -4.4 | 110.7 | -7.0 | -2.6 |
| **20^1^ - CH_3_** | 19.9 | 6.1 | 13.8 | 11.5 | 8.4 | -5.4 | 14.3 | 5.6 | 14.9 | 5.0 | -0.6 |
| **F1 - CH_2_** | 60.6 | 60.3 | 0.3 | 57.0 | 3.6 | 3.3 | 58.8 | 1.8 | 63.2 | -2.6 | -4.4 |
| **F2 - CH** | 118.8 | 118.6 | 0.2 | 117.3 | 1.5 | 1.3 | 127.3 | -8.5 | 127.1 | -8.3 | 0.2 |
| **F3 - C** | 140.2 | 153.4 | -13.2 | 144.8 | -4.6 | 8.6 | 146.1 | -5.9 | 142.5 | -2.3 | 3.6 |
| **F3^1^ - CH_3_** | 14.9 | 12.7 | 2.2 | 9.4 | 5.5 | 3.3 | 7.1 | 7.8 | 8.2 | 6.7 | -1.1 |
| **F4 - CH_2_** | 39.1 | 32.6 | 6.5 | 37.4 | 1.6 | -4.9 | 38.7 | 0.4 | 42.8 | -3.7 | -4.1 |
| **F5 - CH_2_** | 26.0 | 27.6 | -1.6 | 11.2 | 14.8 | 16.3 | 24.1 | 1.9 | 25.2 | 0.8 | -1.1 |
| **F6 - CH** | 123.1 | 120.7 | 2.5 | 127.5 | -4.3 | -6.8 | 128 | -4.9 | 129.7 | -6.6 | -1.7 |
| **F7 - C** | 135.8 | 140.4 | -4.5 | 124.5 | 11.4 | 15.9 | 143.2 | -7.4 | 146.4 | -10.6 | -3.2 |
| **F7^1^ - CH_3_** | 14.6 | 13.7 | 0.8 | 11.3 | 3.3 | 2.5 | 10.1 | 4.5 | 4.5 | 10.1 | 5.6 |
| **F8 - CH_2_** | 39.1 | 35.7 | 3.4 | 28.7 | 10.4 | 7.0 | 41.2 | -2.1 | 40.6 | -1.5 | 0.6 |
| **F9 - CH_2_** | 25.0 | 30.3 | -5.3 | 15.6 | 9.3 | 14.7 | 27.7 | -2.7 | 21.5 | 3.5 | 6.2 |
| **F10 - CH** | 123.3 | 112.3 | 11.1 | 112.9 | 10.5 | -0.6 | 131.4 | -8.1 | 131.1 | -7.8 | 0.3 |
| **F11 - C** | 129.5 | 133.8 | -4.3 | 128.2 | 1.3 | 5.6 | 138.1 | -8.6 | 139.5 | -10.0 | -1.4 |
| **F11^1^ - CH_3_** | 16.8 | 15.4 | 1.3 | 9.9 | 6.9 | 5.5 | 23.3 | -6.6 | 9.5 | 7.3 | 13.8 |
| **F12 - CH_3_** | 24.6 | 21.3 | 3.3 | 7.5 | 17.1 | 13.8 | 19.1 | 5.5 | 19 | 5.6 | 0.1 |
| **RMSD** |  |  | 8.4 |  | 8.3 |  | 5.8 |  | 5.9 |  |  |

[a] Experimental data from this work.

[b] Chemical shifts were referenced judging by the least deviation from the experimental data. The reference number for alternating *syn* and *anti* stacks was 165 and for the antiparallel dimers 164. In case of doublings the experimental shift closest to the calculated was included in the table.

**Table S8** ^1^H chemical shifts for the atoms of the *syn* and *anti* BChls *c* in alternating *syn* and *anti* stacks and antiparallel dimers, calculated by CASTEP. Deviations with the experimental data are calculated as σ_s_^C^ expt - σ_s_^C^calc. Deviations with the experimental data are calculated as *σ_s_^H^_expt_* - *σ_s_^H^_calc_*. Deviations between the calculated chemical shifts of *syn* and *anti* BChls are *σ_s_^H^_cal_* (*syn*)- *σ_s_^H^_cal_* (*anti*)

| **Position** | **Data^[a]^** | **Calculated for the alternating *syn* and *anti* stacks** | | | | | **Calculated for the antiparallel dimers** | | | | |
| --- | --- | --- | --- | --- | --- | --- | --- | --- | --- | --- | --- |
|  | ***σ_s_^H^_expt_***  **BChl** | $\boldsymbol{\sigma}_{\boldsymbol{s}}^{\boldsymbol{H}}$***_cal_* (*syn)***  **^[b]^** | ***σ_s_^H^_expt_*- *σ_s_^H^_cal_* (*syn*)** | $\boldsymbol{\sigma}_{\boldsymbol{s}}^{\boldsymbol{H}}$***_cal_***  **(*anti)***  **^[b]^** | ***σ_s_^H^_expt_*- *σ_s_^H^_cal_* (*anti*)** | ***σ_s_^H^_cal_* (*syn*)- *σ_s_^H^_cal_* (*anti*)** | $\boldsymbol{\sigma}_{\boldsymbol{s}}^{\boldsymbol{H}}$***_cal_* (*syn)***  **^[b]^** | ***σ_s_^H^_expt_*- *σ_s_^H^_cal_* (*syn*)** | $\boldsymbol{\sigma}_{\boldsymbol{s}}^{\boldsymbol{H}}$***_cal_***  **(*anti)***  **^[b]^** | ***σ_s_^H^_expt_*- *σ_s_^H^_cal_* (*anti*)** | ***σ_s_^H^_cal_* (*syn*)- *σ_s_^H^_cal_* (*anti*)** |
| **2^1^b - H_3_** | 0.2 | -1.1 | 1.2 | -2.9 | 3.1 | 1.9 | 1.1 | -1.0 | 0.8 | -0.7 | 0.3 |
| **3^1^a - H** | 2.6 | -0.6 | 3.3 | -1.8 | 4.5 | 1.2 | 5.9 | -3.0 | 6.5 | -3.6 | -0.6 |
| **3^2^b - H_3_** | 0.0 | -4.9 | 4.9 | -4.6 | 4.7 | -0.3 | -0.3 | 0.3 | 0.4 | -0.3 | -0.7 |
| **5b - H** | 6.3 | 3.5 | 2.8 | 4.3 | 2.0 | -0.8 | 5.9 | 0.4 | 6.5 | -0.2 | -0.6 |
| **7^1^b - H_3_** | 2.4 | 0.4 | 2.0 | 1.5 | 0.9 | -1.1 | 1.9 | 0.5 | 2.4 | 0.0 | -0.5 |
| **8^1^ - H_2_** | 2.0 | 1.4 | 0.6 | 2.1 | -0.1 | -0.8 | 2.9 | -0.9 | 2.4 | -0.4 | 0.5 |
| **8^2^ - H_3_** | 0.5 | -0.3 | 0.8 | -0.1 | 0.6 | -0.2 | 1.5 | -1.0 | 1.1 | -0.6 | 0.4 |
| **10 - H** | 8.8 | 5.3 | 3.5 | 6.3 | 2.5 | -1.0 | 8.9 | -0.1 | 9.0 | -0.3 | -0.2 |
| **12^1^ - H_2_** | 1.0 | -2.9 | 3.9 | -2.3 | 3.4 | -0.6 | 3.4 | -2.4 | 3.2 | -2.2 | 0.2 |
| **12^2^ - H_3_** | 0.1 | -4.9 | 4.9 | -5.0 | 5.1 | 0.1 | 0.9 | -0.8 | 0.8 | -0.8 | 0.0 |
| **13^2^ - H_2_** | 3.8 | 1.4 | 2.4 | 1.3 | 2.4 | 0.1 | 2.4 | 1.4 | 2.3 | 1.5 | 0.1 |
| **17 - H** | 3.4 | 0.5 | 3.0 | 2.8 | 0.6 | -2.4 | -3.1 | 6.6 | 2.3 | 1.1 | -5.5 |
| **17^1^ - H_2_** | 2.2 | 1.0 | 1.2 | 0.2 | 2.0 | 0.8 | -1.8 | 4.0 | -5.0 | 7.2 | 3.2 |
| **17^2^ - H_2_** | 2.0 | 1.8 | 0.2 | 1.3 | 0.7 | 0.5 | 1.7 | 0.3 | -5.2 | 7.1 | 6.9 |
| **18a - H** | 3.5 | 3.5 | 0.0 | 3.6 | -0.1 | -0.1 | 2.8 | -1.4 | 2.7 | -1.4 | 0.1 |
| **18^1^a - H_3_** | 0.7 | -0.3 | 0.9 | 0.5 | 0.2 | -0.8 | -5.0 | -1.3 | 0.8 | -7.0 | -5.7 |
| **20^1^ - H_3_** | 2.3 | 1.0 | 1.3 | 1.7 | 0.6 | -0.7 | 1.8 | 0.4 | 2.3 | 0.0 | -0.4 |
| **RMSD** |  |  | 2.7 |  | 2.5 |  |  | 2.2 |  | 3.2 |  |

[a] Experimental data from this work.

[b] Chemical shifts were referenced judging by the least deviation from the experimental data. The reference number for alternating *syn* and *anti* stacks was 23 and for the antiparallel dimers was 30. In case of doublings the experimental shift closest to the calculated was included in the table.

Stabilization of the BChl c aggregate

To understand, which energy contributions are responsible for joining the stacks together, we calculated the energies of one, two and three connected stacks, limited to 6 BChls per stack (Table S9) without boundary conditions. When scaled by the number of BChls, for all parallel cells (packing modes 1, 2 and alternating *syn* and *anti* stacks) the increase of the non-bonding energy was more than proportional. Especially the electrostatic terms showed dependency on the extent of overlap in the structure, which contributes to the converging evidence that the π−π stacking interactions primarily drive the aggregate formation (Ganapathy et al. 2009b) due to cooperative effects. For all packing modes hydrogen bonding has a small influence, less or near 1 eV. Van der Waals forces on contrary destabilize the packing.

**Table S9** Energy contributions for the different number of stacks in parallel packing modes. Energy was dissected using the modified Dreiding forcefield, after a full unit cell optimization with CASTEP. Every stack has 6 BChls, the top of which is coordinated by a H_2_O molecule. Boundary conditions were removed. Although the force field calculations are intrinsically less accurate than the CASTEP, general trends emerge regarding the stabilization of the chlorosome model structure. For alternating *syn* and *anti* stacks, stacks of all-*syn* and all-*anti* BChls alternate and are thus abbreviated as S or A, resulting in different combinations: SAS, ASA, AS, SA.

|  | **Packing mode1** | | | **Packing mode 2** | | | **Alternating *syn* and *anti* stacks** | | | | | |
| --- | --- | --- | --- | --- | --- | --- | --- | --- | --- | --- | --- | --- |
| **Number of stacks** | **3** | **2** | **1** | **3** | **2** | **1** | **3 - SAS** | **3 - ASA** | **2 - AS** | **2 - SA** | **1 - A** | **1 - S** |
| **Total energy** | 265.6 | 184.7 | 101.2 | 273.2 | 187.9 | 101.4 | 261.2 | 260.1 | 183.4 | 180.6 | 100.2 | 99.0 |
| **Contributions to total energy (eV):** | | |  |  |  |  |  |  |  |  |  |  |
| **Valence energy (diag. terms)** | 270.7 | 180.5 | 90.2 | 270.5 | 180.5 | 90.2 | 285.2 | 282.2 | 189.1 | 189.1 | 96.0 | 93.1 |
| **Bond** | 70.7 | 47.1 | 23.6 | 64.5 | 43.1 | 21.6 | 64.6 | 65.6 | 43.4 | 43.4 | 21.2 | 22.2 |
| **Angle** | 173.8 | 115.9 | 57.9 | 179.2 | 119.4 | 59.7 | 178.5 | 179.4 | 119.3 | 119.3 | 59.2 | 60.1 |
| **Torsion** | 16.5 | 11.0 | 5.5 | 20.1 | 13.4 | 6.7 | 27.9 | 23.1 | 17.0 | 17.0 | 10.9 | 6.1 |
| **Inversion** | 9.7 | 6.5 | 3.2 | 6.7 | 4.5 | 2.2 | 14.2 | 14.1 | 9.4 | 9.4 | 4.8 | 4.7 |
| **Non-bond energy** | -5.0 | 4.2 | 10.9 | 2.7 | 7.4 | 11.2 | -24.0 | -22.1 | -5.8 | -8.5 | 4.2 | 5.9 |
| **Hydrogen bond** | -0.9 | -0.4 | 0.0 | -0.6 | -0.3 | 0.0 | -0.6 | -0.6 | 0.0 | -0.6 | 0.0 | 0.0 |
| **van der Waals** | 52.5 | 36.3 | 20.1 | 54.4 | 37.2 | 19.9 | 34.6 | 36.8 | 26.1 | 24.1 | 13.2 | 15.3 |
| **Electrostatic** | -56.7 | -31.6 | -9.1 | -51.1 | -29.5 | -8.7 | -57.9 | -58.3 | -31.8 | -32.0 | -9.0 | -9.3 |

HCH spectra


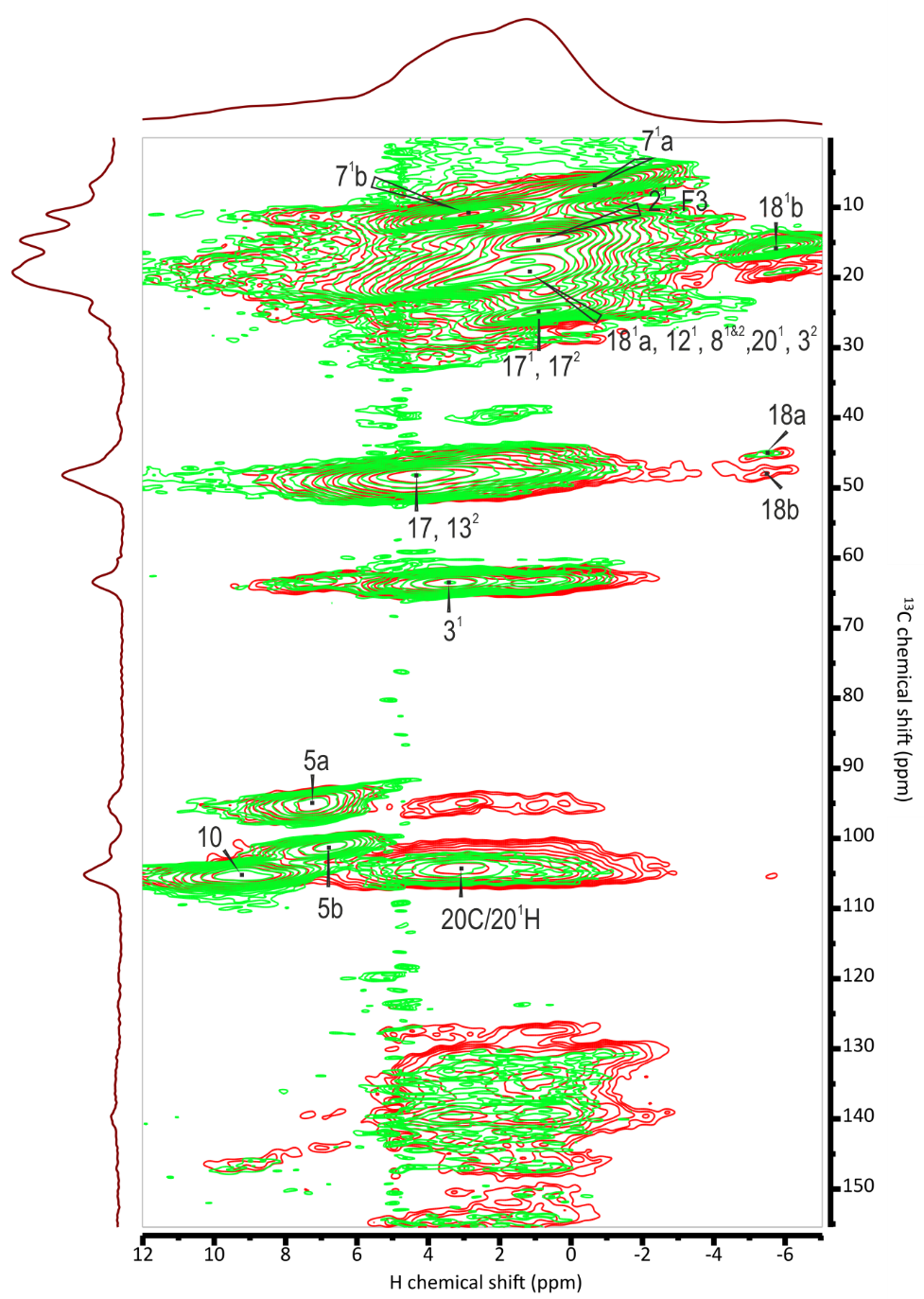


**Fig. S7** Detailed representation of the ^1^H-^13^C HCH spectra of WT *Cba*. *tepidum* represented at high resolution compared to the composite overview in Fig. 3B. HCH with 0.5 (red) and 3 (green) ms mixing times measured at the 800 MHz magnet with 50 kHz MAS. The major and minor doubling components are marked with a and b. They can be attributed to H-bonded and non-H-bonded BChls, respectively. The proton signal at around -6 ppm has been assigned to 18^1^b, because it appears as a doublet and correlates with both fractions of C-18.

References

Adams PG, Cadby AJ, Robinson B, Tsukatani Y, Tank M, Wen J, Blankenship RE, Bryant DA, Hunter CN (2013) Comparison of the physical characteristics of chlorosomes from three different phyla of green phototrophic bacteria. Biochimica et Biophysica Acta - Bioenergetics 1827 (10):1235-1244. doi:<https://doi.org/10.1016/j.bbabio.2013.07.004>

Balaban TS, Holzwarth AR, Schaffner K, Boender GJ, de Groot HJM (1995) CP-MAS ^13^C-NMR dipolar correlation spectroscopy of ^13^C enriched chlorosomes and isolated bacteriochlorophyll *c* aggregates of *Chlorobium tepidum*: The self-organization of pigments is the main structural feature of chlorosomes. Biochemistry 34:15259-15266

Chow HC, Serlin R, Strouse CE (1975) Crystal and molecular structure and absolute configuration of ethyl chlorophyllide  *a*-dihydrate. A model for the different spectral forms of chlorophyll *a* Journal of the American Chemical Society 97:7230-7237. doi:<https://doi.org/10.1021/ja00858a006>

Ganapathy S, Oostergetel GT, Reus M, Tsukatani Y, Chew AGM, Buda F, Bryant DA, Holzwarth AR, de Groot HJM (2012) Structural variability in wild-type and *bchQ bchR* mutant chlorosomes of the green sulfur bacterium *Chlorobaculum tepidum*. Biochemistry 51 (22):4488-4498. doi:<https://doi.org/10.1021/bi201817x>

Ganapathy S, Oostergetel GT, Wawrzyniak PK, Reus M, Gomez Maqueo Chew A, Buda F, Boekema EJ, Bryant DA, Holzwarth AR, de Groot HJM (2009a) Alternating *syn-anti* bacteriochlorophylls form concentric helical nanotubes in chlorosomes. ProcNatl AcadSci 106 (21):8525-8530. doi:<https://doi.org/10.1073/pnas.0903534106>

Ganapathy S, Sengupta S, Wawrzyniak PK, Huber V, Buda F, Baumeister U, Würthner F, de Groot HJM (2009b) Zinc chlorins for artificial light-harvesting self assemble into antiparallel stacks forming a microcrystalline solid-state material. ProcNatl AcadSci 106:11472-11477. doi:<https://doi.org/10.1073/pnas.0811872106>

Gomez Maqueo Chew A, Frigaard NU, Bryant DA (2007) Bacteriochlorophyllide *c* C-8^2^ and C-12^1^ methyltransferases are essential for adaptation to low light in *Chlorobaculum tepidum*. The Journal of Bacteriology 189:6176-6184. doi:<https://doi.org/10.1128/JB.00519-07>

Jaroniec CP Dipole distance calculator. <https://chemistry.osu.edu/~jaroniec/nmr/calcdist.php>.

Li X, Buda F, de Groot HJM, Sevink GJA (2018) Contrasting modes of self-assembly and hydrogen-bonding heterogeneity in chlorosomes of *Chlorobaculum tepidum*. The Journal of Physical Chemistry C 122 (26):14877-14888. doi:<https://doi.org/10.1021/acs.jpcc.8b01790>

Mehring M (1983) M. Mehring. Principles of High Resolution NMR in Solids. Springer-Verlag, Berlin, Heidelberg, New York, 1983. 342 pp. Cloth $71.80. ISBN 3-540-11852-7, vol 21. Organic Magnetic Resonance, vol 12. doi:<https://doi.org/10.1002/omr.1270211211>

Oostergetel GT, Reus M, Gomez Maqueo Chew A, Bryant DA, Boekema EJ, Holzwarth AR (2007) Long-range organization of bacteriochlorophyll in chlorosomes of Chlorobium tepidum investigated by cryo-electron microscopy. FEBS Letters 581 (28):5435-5439

Tian Y, Camacho R, Thomsson D, Reus M, Holzwarth AR, Scheblykin IG (2011) Organization of bacteriochlorophylls in individual chlorosomes from Chlorobaculum tepidum studied by 2-dimensional polarization fluorescence microscopy. Journal Of The American Chemical Society 133 (43):17192-17199. doi:10.1021/ja2019959

van Rossum BJ, de Groot CP, Ladizhansky V, Vega S, H.J.M. dG (2000) A method for measuring heteronuclear (^1^H−^13^C) distances in high speed MAS NMR. Journal of the American Chemical Society 122 (14):3465-3472. doi:<https://doi.org/10.1021/ja992714j>

van Rossum BJ, Steensgaard DB, Mulder FM, Boender GJ, Schaffner K, Holzwarth AR, de Groot HJM (2001) A refined model of the chlorosomal antennae of the green bacterium Chlorobium tepidum from proton chemical shift constraints obtained with high-field 2-D and 3-D MAS NMR dipolar correlation spectroscopy. Biochemistry 40 (6):1587-1595. doi:10.1021/bi0017529

Author Contributions

Yuliya A. Miloslavina: writing, figures, tables, references, MAS NMR measurements and data analysis in Leiden and in Göttingen, construction and analysis of repeat units and packing modes in the Materials Studio, theoretical calculations and analysis, EM data modeling, construction of the tubes in Crystal Maker.

Brijith Thomas: support with Castep calculations

Michael Reus: sample preparation

Karthick Babu Sai Sankar Gupta: MAS NMR measurements setup help in Leiden

Gert T. Oostergetel: provided original EM spectra and explain how to do EM modeling.

Loren Andreas: supervised MAS NMR measurements in Göttingen, writing-editing

Alfred R. Holzwarth: discussions about optical spectroscopy, modeling was first started by modifications of Alfred’s model

Huub J.M. de Groot: coordination of the project, writing, editing
